# LSD1 co-repressor Rcor2 orchestrates neurogenesis in the developing mouse brain

**DOI:** 10.1038/ncomms10481

**Published:** 2016-01-22

**Authors:** Yixuan Wang, Qian Wu, Peng Yang, Chenfei Wang, Jing Liu, Wenyu Ding, Wensu Liu, Ye Bai, Yuanyuan Yang, Hong Wang, Shaorong Gao, Xiaoqun Wang

**Affiliations:** 1 Clinical and Translational Research Center of Shanghai First Maternity and Infant Health Hospital, School of Life Sciences and Technology, Tongji University, Shanghai 200092, China; 2 State Key Laboratory of Brain and Cognitive Science, CAS Center for Excellence in Brain Science and Intelligence Technology, Beijing MRI Centre for Brain Research, Institute of Biophysics, Chinese Academy of Sciences, Beijing Institute for Brain Disorders, 8803, 8th Building, 15 Datun Road, Chaoyang District, Beijing 100101, China; 3 National Institute of Biological Sciences (NIBS), Beijing 102206, China

## Abstract

Epigenetic regulatory complexes play key roles in the modulation of transcriptional regulation underlying neural stem cell (NSC) proliferation and progeny specification. How specific cofactors guide histone demethylase LSD1/KDM1A complex to regulate distinct NSC-related gene activation and repression in cortical neurogenesis remains unclear. Here we demonstrate that Rcor2, a co-repressor of LSD1, is mainly expressed in the central nervous system (CNS) and plays a key role in epigenetic regulation of cortical development. Depletion of Rcor2 results in reduced NPC proliferation, neuron population, neocortex thickness and brain size. We find that Rcor2 directly targets Dlx2 and Shh, and represses their expressions in developing neocortex. In addition, inhibition of Shh signals rescues the neurogenesis defects caused by Rcor2 depletion both *in vivo* and *in vitro*. Hence, our findings suggest that co-repressor Rcor2 is critical for cortical development by repressing Shh signalling pathway in dorsal telencephalon.

## Abstract (web summary)

Epigenetic regulation plays a key role in cortical development. Here the authors show that Rcor2, a co-repressor of the histone demethylase LSD1/KDM1A complex, regulates neural progenitor cell proliferation and cortical neurogenesis by repressing sonic hedgehog signaling.

## Graphical abstract

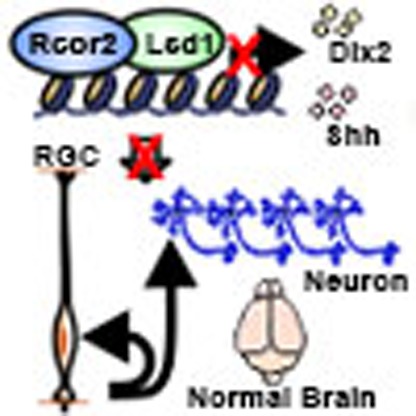

Previous studies have defined neural stem cell (NSC) and Neural Progenitor Cell (NPC) populations in the developing neuroepithelium of the rodent neocortex with region-specific neuronal progenies generated at embryonic stages. Whereas cortical projection excitatory neurons derive from radial glial cells (RGCs) at the dorsal (pallial) telencephalon and migrate radially into the cortical plate, neocortical interneurons are generated in the ventral telencephalon, including the ganglionic eminences and the preoptic area (PoA), and migrate tangentially over long distances to reach the neocortex^1^,^2^,^3^,^4^,^5^,^6^,^7^,^8^. During embryonic brain development, a series of morphogens are involved in the establishment of patterning in the neuroepithelum, which provide specific environments to form different substructures of functional brain. Multiple studies exploring the mechanism of temporal and spatial regulation that lead to the remarkable cellular diversity of the mature neocortex have underlined the importance of organizing signals such as Sonic hedgehog (Shh), fibroblast growth factors, WNTs, and bone morphogenetic proteins, which provide positional information in body axes^7^,^9^,^10^,^11^,^12^,^13^,^14^. Among these signals, Shh expression is first observed in the mesendoderm and diencephalon between E8.5 and E9 during telencephalic development, which are then restricted to the Medial Ganglionic Eminence (MGE) and PoA by E9.5 and by E12.5, respectively, to pattern the ventral brain, while maintaining a very low activity in the developing dorsal cortex^15^,^16^,^17^,^18^,^19^. However, how Shh signal is controlled and restricted to the specific regions and how Shh regulates NSC/NPC proliferation and differentiation still remains poorly understood.

Extensive studies have suggested that NSC differentiation and fate specification is regulated by a series of intrinsic and extrinsic signals. Although the mechanisms of regional restriction and fate determination of NSCs are not yet fully understood, it is becoming apparent that intrinsic epigenetic regulation is closely involved through DNA methylation, histone modification and non-coding RNA expression^20^,^21^,^22^,^23^,^24^,^25^,^26^. Histone demethylase LSD1, also known as KDM1A, is a key component of various protein complexes that contain transcription co-repressors, including Rcor1 (CoREST), BHC80, HDAC1/2, CtBP, BRAF35 and several zinc finger proteins^26^,^27^,^28^. As the first identified RCOR protein, Rcor1 is widely studied in various types of cells, playing important roles in mouse erythropoiesis^29^ and neuronal or synaptic gene regulation by modifying chromatin configuration^30^,^31^,^32^,^33^. Sharing 70% sequence similarities with Rcor1, Rcor2 can form a protein complex with LSD1, to facilitate its demethylase activity on nucleosome in embryonic stem cells (ESCs)^34^. Furthermore, Rcor2 but not Rcor1 has been described as a Sox2 substitute to regulate ESC proliferation and promote the formation of induced pluripotent stem cells^34^. However, the *in vivo* function of Rcor2 in development is unknown.

Here we demonstrate that Rcor2 is mainly expressed in the central nervous system (CNS) and affects NSC proliferation and neurogenesis during cortical development. We find that Rcor2 associating with LSD1 plays a determinant role in directly binding to Dlx2 and Shh promoter regions, to inhibit gene expression. The disruption of Shh, the direct regulation target of Rcor2 and upstream effector of Dlx2, rescues the neurogenesis defects caused by Rcor2 depletion, suggesting that Rcor2 plays a critical role of temporal and spatial regulation of gene expressions to safeguard cortical neurogenesis in brain development.

## Results

### Rcor2 is mainly expressed in the CNS

To analyse Rcor2's function and mechanism during development, *Rcor2* gene-trapped mice (Rcor2^LacZ^) were used as described in detail methods^35^. A *LacZ* reporter gene cassette, flanked by two FRT sequences, was introduced before the first exon of the Rcor2 gene locus, and first several exons were flanked by two *LoxP* sites in Rcor2^LacZ^ mice (Supplementary Fig. 1a). With these Rcor2^LacZ^ mice, we generated the wild-type Rcor2^flox/flox^ (Rcor2^fl/fl^) mice by Flippase splicing. After this, we were able to investigate conditional loss-of-function of Rcor2 (Rcor2^cko^) in specific tissues on Cre recombination in Rcor2^fl/fl^ mice (Supplementary Fig. 1a).

**Figure 1:**
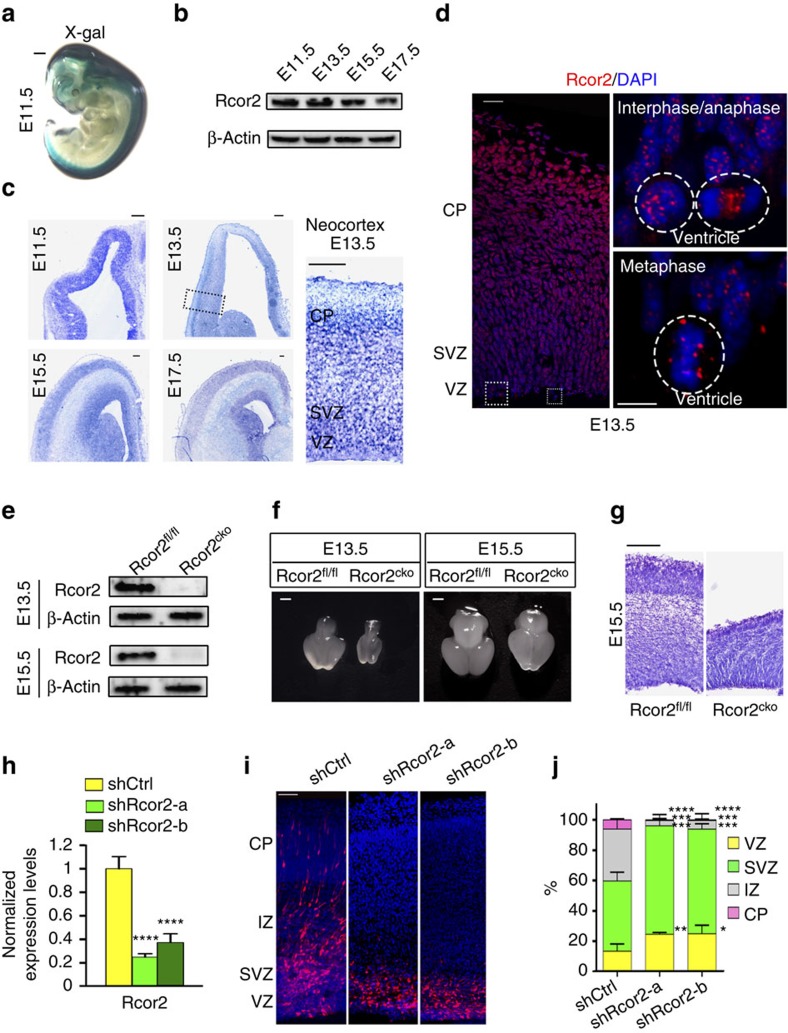
Rcor2 expresses in the CNS and regulates cortical development. (**a**) X-gal staining to detect Rcor2 expression patterns. Whole-embryo staining at E11.5 stage shows Rcor2 is mainly expressed in the CNS. Scale bar, 1 mm. (**b**) Western blot analysis of Rcor2 expression levels during brain development. The decreased expression of Rcor2 with embryonic development is noteworthy. β-Actin is used as an endogenous control. (**c**) *In situ* hybridization to detect endogenous *Rcor2* mRNA expression patterns in cortical development at E11.5, E13.5, E15.5 and E17.5. Insets show high-magnification image of Rcor2 expression in the neocortex at E13.5. VZ, ventricular zone; SVZ, subventricular zone; CP, cortical plate. Scale bar, 100 μm. (**d**) Confocal images of immunofluorescence to detect cellular localization of Rcor2 in the neocortex at E13.5. Rcor2 localized mainly in the nucleus at interphase and metaphase, and localized between separated chromosomes in anaphase of dividing cells in VZ. Dotted lines circle the shape of nuclei. Scale bar, 5 μm. (**e**) Western blot analysis of Rcor2 expression level in Rcor2^fl/fl^ and Rcor2^cko^ brains at E13.5 and E15.5, respectively. Rcor2 expression was depleted in Rcor2^cko^ brains. β-Actin is used as an endogenous control. (**f**) Representative images of Rcor2^fl/fl^ and Rcor2^cko^ brain size at different stages of development. Rcor2^cko^ mice show severe brain growth retardation at E13.5 and E15.5. Scale bar, 1 mm. (**g**) Representative images of Rcor2^fl/fl^ and Rcor2^cko^ cortex at E15.5 by Nissl staining. Structural abnormalities of lamination with reduced cortical thickness are observed in Rcor2^cko^ cortex. Scale bar, 200 μm. (**h**) RT–qPCR analysis of knockdown efficiencies of the two shRNAs targeting Rcor2. Transcripts were normalized to the control group. Data are shown as mean±s.e.m., *t*-test, ****P<0.0001, *n*=3. (**i**) Confocal images of E16.5 cortical sections electroporated with shControl (red), shRcor2-a (red) and shRcor2-b (red) plasmids at E13.5. Knockdown of Rcor2 results in impaired cortical development. IZ, intermediate zone. Scale bar, 20 μm. (**j**) Quantification of the percentage of RFP^+^ cells in different regions of the developing neocortex after electroporation as shown in **i**. proportion of RFP^+^ cells in different zones (*y* axis). Data are shown as mean±s.e.m., *t*-test, *P<0.05, **P<0.01, ***P<0.001 and ****P<0.0001, *n*=3 individual experiments.

First, by LacZ enzymatic activity assay, we examined the expression and localization of Rcor2 during embryonic development. X-gal staining of Rcor2^LacZ/+^ embryos at embryonic day 11.5 (E11.5) revealed Rcor2 was mainly and highly expressed in the CNS, including the brain and spinal cord (Fig. 1a), indicating that Rcor2 may play an important role in the neural development of embryos. Western blot analysis of Rcor2 expression levels revealed decreased expression of Rcor2 in embryonic brains during development, suggesting Rcor2 may primarily function in early brain development (Fig. 1b). To examine the endogenous Rcor2 expression pattern during cortical development, we performed *in situ* hybridization and immunostaining by an RNA probe or antibody, and both were specific by no signals detected in the neural lineage-knockout Rcor2^fl/fl^Nes^Cre^ (Rcor2^cko^) cortex (Supplementary Fig. 1b,c). At early stages of brain development, Rcor2 messenger RNA and protein levels were high in mostly all cells in the wild-type mice cortex (Fig. 1c,d). Similar to the protein expression pattern as we observed in western blotting results (Fig. 1b), *Rcor2* mRNA levels were also decreased as brains developed (Fig. 1c). As a transcriptional co-repressor, Rcor2 localized mainly in the cell nucleus, as expected (Fig. 1d). Interestingly, Rcor2 exhibited diverse subcellular localizations at different stages of the cell cycle, suggesting that it might directly play a role in cell division process. We observed Rcor2 protein puncta in the nucleus in interphase RG cells and localized mainly in chromosomes in metaphase RGCs in the ventricular zone (VZ). When cells entered anaphase, Rcor2 partially translocated to the space between two sets of separated chromosomes (Fig. 1d). Our observations are similar to the localization patterns of histone demethylase LSD1 in ESCs in previous studies, which showed LSD1 can regulate short timescale gene expressions during cell cycle progression by being recruited to or displaced from chromatin^36^, suggesting that Rcor2 may directly regulate NSC or NPC divisions by associating with LSD1. Taken together, Rcor2 is dominantly expressed in the CNS during mouse development and its expression pattern in the NSCs or NPCs exhibits regional differences in cell cycle, suggesting that Rcor2 may play an important role in regulating cerebral cortex development.

### Rcor2 regulates neurogenesis during cortical development

Next, we adopted Rcor2^cko^ mice to study Rcor2's role by conditional loss-of-function in neural lineage (Supplementary Fig. 1a). Western blot analysis of brains at E13.5 and E15.5 confirmed the expression of Rcor2 is completely depleted in Rcor2^cko^ brains (Fig. 1e). Investigation of the Rcor2^cko^ embryos revealed that the brain sizes were greatly decreased compared with the control (Rcor2^fl/fl^) embryos at respective developmental stages (Fig. 1f). Representative Nissl staining of Rcor2^fl/fl^ and Rcor2^cko^ brains slices at different developmental stages exhibited not only significantly reduced thickness of the cortex but also structural abnormalities of layers on Rcor2 depletion (Fig. 1g and Supplementary Fig. 1d,e), suggesting that depletion of Rcor2 affects neocortical development.

For further confirmation, *in-utero* electroporation of two red fluorescent protein (RFP)–short hairpin RNAs (shRNAs) targeting different sites of *Rcor2* mRNA were performed at the lateral ventricle in brains at E13.5, respectively, both of which could efficiently reduce Rcor2 expression level by 70% compared with the control group (Fig. 1h). Disruption of Rcor2 resulted in significant accumulation of RFP^+^ cells at the VZ and subventricular zone (SVZ) regions, whereas it resulted in a dramatic reduction at the intermediate zone and cortical plate regions at E16.5, indicating impaired cortical development on Rcor2 downregulation (Fig. 1i,j). Moreover, the cortical developmental defects caused by Rcor2 knockdown could be rescued by electroporation of *Rcor2* overexpression plasmid, but not *Rcor1* expression, the homologue of Rcor2, which plays important roles in pyramidal cortical neuron development as previously reported (Supplementary Fig. 1f,g)^30^. All the results above suggest that Rcor2 directly plays a critical role in regulating embryonic cortical development, which is distinguishable from Rcor1.

### Rcor2 regulates cortical NSC/NPC proliferation

**Figure 2:**
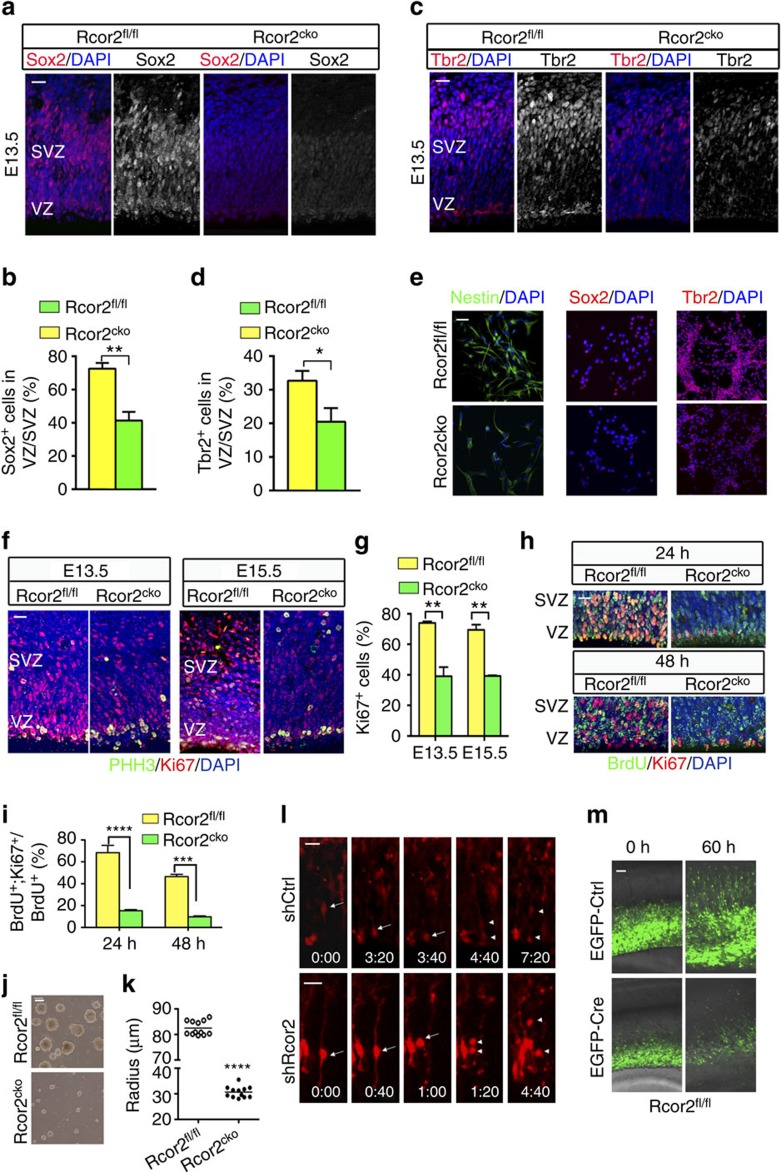
Rcor2 regulates cortical NSC/NPC population and proliferation during development. (**a**) Immunostaining images of Sox2 at E13.5. Sox2 is dramatically reduced on Rcor2 depletion. VZ, ventricular zone; SVZ, subventricular zone. Scale bar, 20 μm. (**b**) Quantification of Sox2^+^ cell ratios in VZ/SVZ regions shown in **a**. Data are shown as mean±s.e.m., *t*-test, ** *P*<0.01, *n*=3 individual experiments. (**c**) Confocal images of Tbr2 expression at E13.5. Tbr2 is dramatically reduced on Rcor2 depletion. Scale bar, 20 μm. (**d**) Quantification of Tbr2^+^ cell ratios in VZ/SVZ regions shown in **c**, respectively. Data are shown as mean±s.e.m., *t*-test, *P<0.05, *n*=3 individual experiments. (**e**) Immunostaining images of Nestin, Sox2 and Tbr2 in cultured Rcor2^fl/fl^ and Rcor2^cko^ NPCs, all of which exhibit significantly reduced expression in the Rcor2^cko^ NPCs. Scale bar, 20 μm. (**f**) Confocal images of immunofluorescence for Ki67 and PHH3 in Rcor2^fl/fl^ and Rcor2^cko^ cortex at E13.5 and E15.5. Ki67 signals (red), but not PHH3 signals (green), are dramatically reduced in Rcor2^cko^ developing brains. Scale bar, 20 μm. (**g**) Quantification of Ki67^+^ cells in the VZ/SVZ regions of the developing neocortex as shown in **f**. Data are shown as mean±s.e.m., *t*-test, **P<0.01, *n*=3 separate stainings from three independent brains. (**h**) Confocal images of BrdU (green) and Ki67 (red) staining in Rcor2^fl/fl^ and Rcor2^cko^ cortex 24 and 48 h after BrdU incorporation. Scale bar, 100 μm. (**i**) Quantification of the cell cycle exit by percentage of BrdU^+^ and Ki67^+^ NPCs divided by BrdU^+^ cells shown in **h**. Data are shown as mean±s.e.m., *t*-test, ***P<0.001 and ****P<0.0001, *n*=3 individual experiments. (**j**,**k**) Representative images (**j**) and quantification of (**k**) of Rcor2^fl/fl^ and Rcor2^cko^ neurosphere sizes. The neurospheres'radius of Rcor2^cko^ are much smaller than those of Rcor2^fl/fl^, *t*-test, ****P<0.0001, *n*=12. Scale bar, 50 μm. (**l**) Representative time-lapse imaging of the RGC dividing process in the sections of the cerebral cortex electroporated with RFP-shControl (upper panels) and RFP-shRcor2 (lower panels). The radial glial dividing pattern is abnormal on Rcor2 knockdown, resulting in cell death. Arrows, mother RGCs. Arrowheads, two daughter cells. Scale bar, 50 μm. (**m**) Representative time-lapse images of Rcor2^fl/fl^ cortex sections electroporated with EGFP-Control (upper panels) and EGFP-Cre (lower panels). Loss of cells is observed with Rcor2 knockout by Cre recombinase electroporation. Scale bar, 50 μm.

Given the profound effects of Rcor2 on cortical development, we examined the proliferation and neurogenesis capacities of NSCs/NPCs on Rcor2 depletion during cortical development. Compared with the control group, decreased expressions of NPC markers Sox2 and Nestin were observed by immunostaining in Rcor2^cko^ cortex at E13.5 (Fig. 2a,b and Supplementary Fig. 2a). In addition, the population of intermediate progenitor cells (IPCs) identified by Tbr2 staining was also reduced on Rcor2 depletion (Fig. 2c,d). Reverse transcriptase–quantitative PCR (RT–qPCR) analysis using the developing neocortex samples at E13.5 and E15.5 also exhibit similar results (Supplementary Fig. 2b). To further confirm the observations above, we checked the expressions of Nestin, Sox2 and Tbr2 in the *in-vitro*-cultured Rcor2^fl/fl^ and Rcor2^cko^ NPCs established from Rcor2^fl/fl^ and Rcor2^cko^ ESC lines by immunostaining, which were similar to our *in vivo* observations (Fig. 2e). Above all, both *in-vivo* and *in-vitro* results suggest that NPC and IPC populations are significantly affected on Rcor2 knockout, which may result in proliferation defects during cortical development.

Next, we examined why NPC/IPC populations reduced on Rcor2 depletion. Immunostainings followed by quantification of cell proliferation markers Ki67 and phospho-histone H3 (PHH3) were performed in both Rcor2^fl/fl^ and Rcor2^cko^ developing cortex. Although PHH3 signals were not significantly affected, dramatically reduced Ki67 signals were observed in the VZ and SVZ regions on Rcor2 knockout during development (Fig. 2f,g and Supplementary Fig. 2c left panel), indicating that depletion of Rcor2 would significantly affect proliferative cell numbers during cortical development. However, we observed that the mitotic index increased in VZ/SVZ regions in Rcor2^cko^ cortex (Supplementary Fig 2c right panel), which is a result from decrease of Ki67^+^ cells compared with wild type, but no obvious changes of PHH3^+^ cell (Fig. 2g and Supplementary Fig. 2c left panel). For further confirmation, we performed cell cycle exit analysis in Rcor2^fl/fl^ and Rcor2^cko^ brains at E13.5, respectively. Pulse-labelling cortex were analysed 24 or 48 h after 5-bromodeoxyuridine (BrdU) incorporation and Ki67 was used to identify cells that entered the following cell cycles (Fig. 2h). We found that 68.33±6.73% Rcor2^fl/fl^ NPCs and 15.28±1.02% Rcor2^cko^ NPCs in VZ/SVZ were able to undergo further rounds of cell division in the next 24 h. At 48 h after BrdU pulsing, only 9.71±0.84% Rcor2^cko^ NPCs were still in the cell cycles compared with 46.44±2.08% Rcor2^fl/fl^ NPCs (Fig. 2i), suggesting that cortical NPCs exit cell cycle at earlier time with Rcor2 depletion during development.

*In vitro*, the neurosphere sizes of both Rcor2^fl/fl^ and Rcor2^cko^ NPCs were compared, which were significantly reduced on Rcor2 disruption (Fig. 2j,k). To examine the divisions of the cultured NPCs directly, we labelled the Rcor2^fl/fl^ and Rcor2^cko^ NPCs with green fluorescent protein (GFP)-expressing retrovirus (retro-GFP), which infected only dividing cells, and monitored the division process till 98 h after virus infection. To our observation, GFP^+^ cells divided robustly in the wild-type group; however, the GFP^+^ cells increased much more slowly on Rcor2 depletion (Supplementary Fig. 2d,e). We also performed cell cycle exit analysis by BrdU incorporation followed by Ki67 antibody staining in the cultured Rcor2^fl/fl^ and Rcor2^cko^ NPCs, respectively, which also showed earlier cell cycle exit on Rcor2 depletion (Supplementary Fig. 2f,g).

To further dissect Rcor2's role in NPC proliferation regulation, time-lapse imaging of the RGC dividing process was recorded after Rcor2 knockdown by specific shRNAs mentioned above (Fig. 1h). RGCs with Rcor2 knockdown showed interkinetic nuclear migration defect and the daughter cells showed apoptosis after separation (Fig. 2l), suggesting that Rcor2 is required for radial glia maintenance and cell division. In addition, we also knocked out Rcor2 by electroperating EGFP-Cre and EGFP-Control plasmids in Rcor2^fl/fl^ mouse embryos at E13.5 and recorded NPC proliferation in neocortex sections for 60 h, respectively. Compared with the control group in which robust cell proliferation and migration were observed, Rcor2 knockout NPC/IPCs generated fewer daughter cells (Fig. 2m). Immunostaining results showed enhanced Caspase 3 signals among Rcor2^cko^ neocortex compared with Rcor2^fl/fl^ cortex at E15.5 (Supplementary Fig. 2h). Significantly increased cell death was also observed in both cultured NPCs and neurons on Rcor2 knockout by TdT-mediated dUTP nick end labelling assays (Supplementary Fig. 2i and 2j). All together, these results suggest that Rcor2 plays an important role in cortical development via regulating NSC/NPC proliferation and maintenance.

### Rcor2 regulates cortical neurogenesis during development

**Figure 3:**
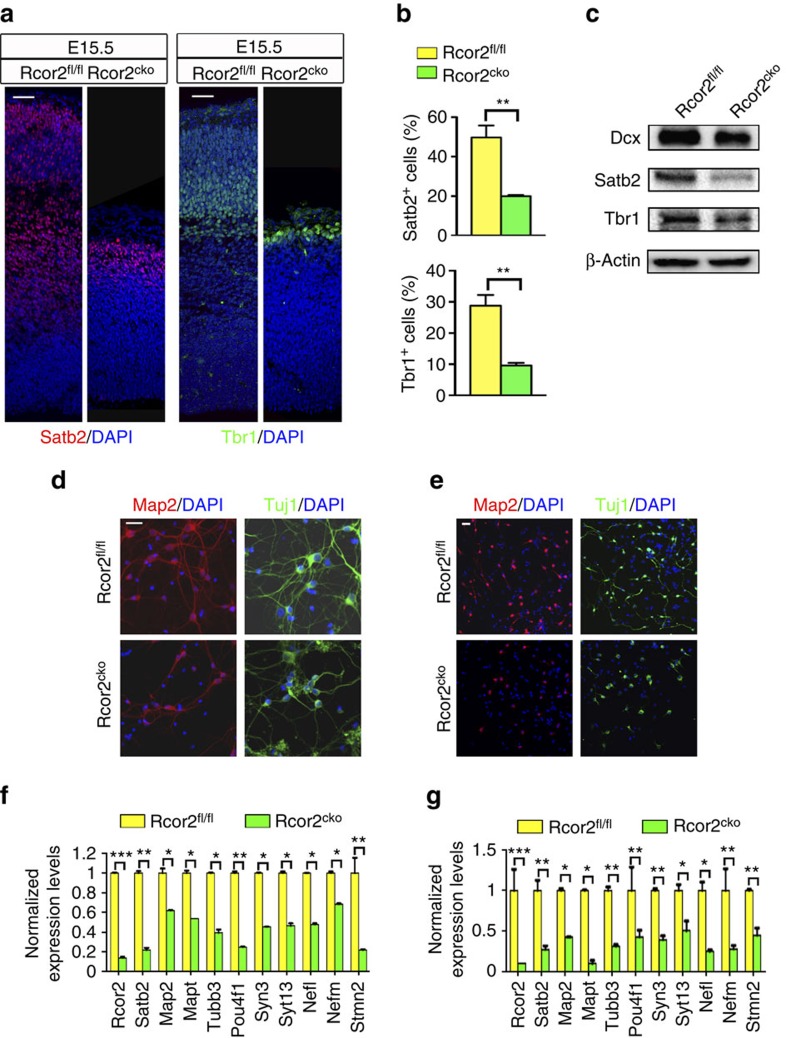
Rcor2 regulates cortical neurogenesis during development. (**a**) Confocal images of Satb2 and Tbr1 expressions in Rcor2^fl/fl^ and Rcor2^cko^ cortex at E15.5, which exhibit significant reduction on Rcor2 knockout. Scale bar, 50 μm. (**b**) Quantification of Satb2^+^ and Tbr1^+^ cells in Rcor2^fl/fl^ and Rcor2^cko^ cortex at E15.5 in **a** indicates Satb2 and Tbr1 expressions are decreased on Rcor2 depletion during development. Data are shown as mean±s.e.m., *t*-test, **P<0.01, *n*=3 individual experiments. (**c**) Western blot to analyse Dcx, Satb2 and Tbr1 expressions in Rcor2^fl/fl^ and Rcor2^cko^ cortex at E15.5. β-Actin is used as an endogenous control. (**d**) Representative images of Map2 and Tuj1 immunostaining in cultured neurons directly dissociated from Rcor2^fl/fl^ and Rcor2^cko^ brain cortex at E15.5. Decreased expression of both markers and reduced neurofilaments can be observed in Rcor2^cko^ cultured neurons. Scale bar, 20 μm. (**e**) Confocal images of *in-vitro* cultured Rcor2^fl/fl^ and Rcor2^cko^ NPCs 5 days post spontaneous differentiation using neuronal marker Map2 and Tuj1 antibodies, both of which are significantly reduced in the differentiated Rcor2^cko^ NPCs. Scale bar, 20 μm. (**f**,**g**) RT–qPCR analysis of neuronal markers expression in both Rcor2^fl/fl^ and Rcor2^cko^ neocortex at E15.5 stage (**f**) and *in-vitro*-cultured Rcor2^fl/fl^ and Rcor2^cko^ NPCs 5 days post spontaneous differentiation (**g**). Transcripts were normalized to Rcor2^fl/fl^ group. Data are shown as mean±s.d., *t*-test, *P<0.05, ** *P*<0.01 and ***P<0.001, *n*=3.

Given the observation of reduced NPC proliferation on Rcor2 depletion, we checked the expressions of neuronal markers in Rcor2^fl/fl^ and Rcor2^cko^ cortex at E15.5. On Rcor2 depletion, a consistent reduction in both Dcx^+^/Satb2^+^/Tbr1^+^ cell numbers and Dcx^+^/Satb2^+^/Tbr1^+^-containing layer thickness was observed during cortical development (Fig. 3a and Supplementary Fig. 3a). Quantification confirmed dramatic decreases of Satb2^+^ and Tbr1^+^ cells, which are postmitotic pyramidal neurons, in Rcor2^cko^ developing neocortex compared with Rcor2^fl/fl^ (Fig. 3b). Western blot analysis also confirmed downregulation of the neuronal markers mentioned above (Fig. 3c), indicating reduction of NPC pool caused by Rcor2 depletion leads to reduced cortical neurons during development. In addition, we also assessed the fate of cells with Rcor2 downregulation by *in-utero* electroporation assay. Three days after injection of RFP–shRNAs targeting Rcor2 mRNA, we found that most RFP^+^ cells localized in VZ/SVZ (Fig. 1i,j) and some of these cells were Pax6^+^ cells (Supplementary Fig. 3b). However, the ratio of Pax6^+^ cells among Rcor2-knockdown cells was greatly reduced compared with control samples (Supplementary Fig. 3c), which was consistent with the results that Rcor2 depletion reduced the NPC pool in mouse developing cortex (Fig. 2a,b). Immunostained with newborn neuron marker Dcx, we found that some of the RFP^+^ cells were Dcx^+^ (Supplementary 3d) and the ratio of Dcx^+^ cells among RFP^+^ cells was also significantly decreased (Supplementary 3e), indicating that Rcor2 depletion impaired neurogenesis, which is consistent with the observation in Rcor2^cko^ mice (Fig. 3).

Immunostaining of Map2 and Tuj1 were also performed in the primary cultured neurons dissociated from Rcor2^fl/fl^ and Rcor2^cko^ cortex at E15.5, which showed greatly decreased expression of both markers and significantly reduced neurofilaments on Rcor2 depletion (Fig. 3d). Similar results were also found in the *in-vitro*-cultured Rcor2^cko^ NPCs subjected to differentiation for 5 days (Fig. 3e). Moreover, RT–qPCR analysis of *in vivo* and *in vitro* samples also exhibited significant downregulation of neuronal markers such as *Map2*, *Tubb3* and *Pou4f1* (Fig. 3f,g). Thus, our data suggest that Rcor2 mediates neurogenesis both *in vivo* and *in vitro*; hence, depletion of Rcor2 leads to affected proliferation of NPC and production of neurons during development.

### Rcor2 regulates neurogenesis as a co-repressor of LSD1

**Figure 4:**
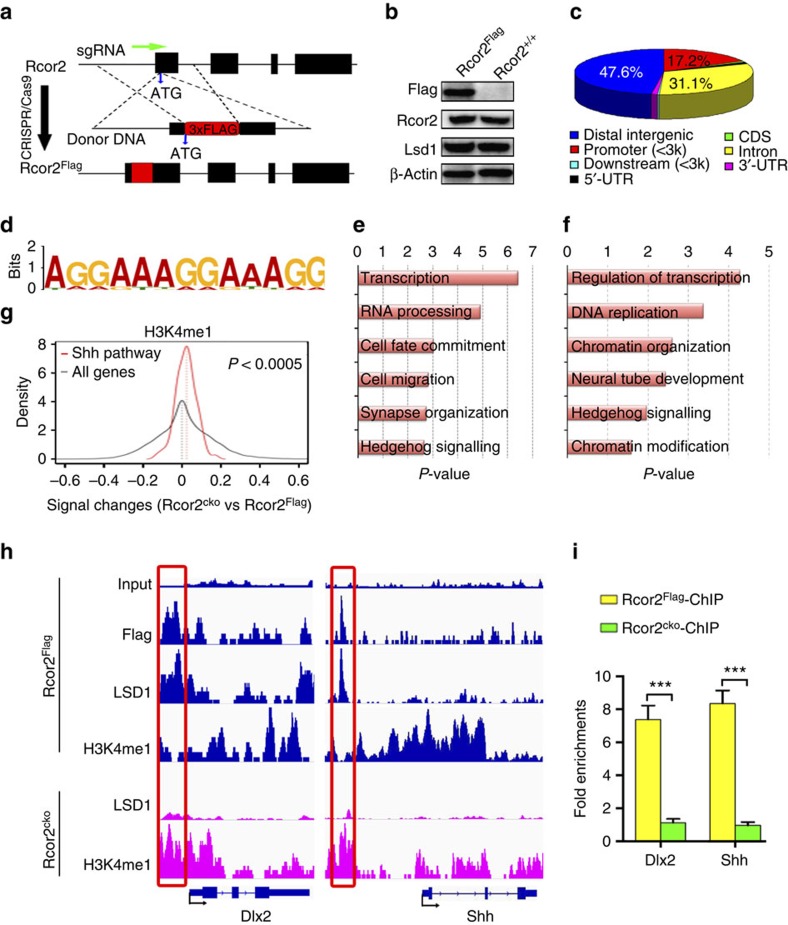
ChIP-seq analysis of Rcor2 enrichments in genome-wide scale reveals direct binding of Rcor2 at regulatory regions of genes related to Shh signalling pathway. (**a**) Schematic overview of strategy to generate an Rcor2^Flag^ knock-in allele by CRISPR/Cas9. The sgRNA sequence site is shown as a green arrowhead. The start codon of Rcor2 is indicated and capitalized. The oligo donor contained 50 bp homologies on both sides flanking the DSB, in which 3 × Flag sequences are labelled as a red box. (**b**) Western blot analysis to validate FLAG, RCOR2 and LSD1 expressions in Rcor2^Flag^ knock-in neocortex using Flag-M2 antibody. β-Actin was used as an endogenous control. (**c**) Pie chart depicts distribution of Rcor2 occupancies in genome-wide scale in FLAG ChIP-seq results using Rcor2^Flag^ knock-in neocortex at E13.5. (**d**) WebLogos of consensus binding motifs of Rcor2 generated by Multiple EM for Motif Elicitation (MEME) motif analysis tool. (**e**) GO analysis for Rcor2-binding regions in genome-wide scale revealed by Flag ChIP-seq results using Rcor2^Flag^ brain. (**f**) GO analysis for LSD1 occupancy in genome-wide scale revealed by LSD1 ChIP-seq results using Rcor2^Flag^ brain. (**g**) Density plots analysis of H3K4me1 signal change in promoter regions (−2- to ∼0.5 kb from TSS) on Rcor2 depletion. Compared with all genes, the change of H3K4me1 signal is significantly (*P*<0.0005, Kolmogorov–Smirnov test) enhanced in the promoter regions of Shh pathway-related genes on Rcor2 depletion. H3K4me1 signal change on Rcor2 depletion (*x* axis); H3K4me1 signal density (*y* axis). (**h**) Gene tracks of Rcor2, LSD1 and H3K4me1 enrichments by ChIP-seq analysis at core promoter regions of Dlx2 and upstream regulatory regions of Shh, which are closely related to Shh signalling. (**i**) ChIP–qPCR analysis of Rcor2^Flag^ and Rcor2^cko^ cortex at E13.5 using specific FLAG-M2 antibody. Significant enrichments of the Rcor2 at the regulatory regions of Dlx2 and Shh gene locus detected in **g** in the Rcor2^flag^ samples are worth noting. Fold enrichments of Rcor2 occupancy compared with input (*y* axis). Data are shown as mean±s.d., *t*-test, ***P<0.001, *n*=3.

Previous studies indicate that Rcor2 works as a co-repressor, closely related to LSD1 complex, to facilitate its nucleosomal demethylase activity^34^. Given the significantly reduced neurogenesis on Rcor2 disruption, we constructed Rcor2^Flag^ knock-in mice in which 3 × *Flag* sequences were introduced behind the start codon of Rcor2 protein by CRISPR/Cas9 system, to identify the targets of Rcor2 during cortical development (Fig. 4a). Western blot analysis confirmed FLAG expression in Rcor2^Flag^ knock-in brains and stable expression of endogenous Rcor2 with a Flag tag (Fig. 4b). Immunostaining of Flag, Rcor2 and Lsd1 antibodies in Rcor2^Flag^ cortex at E11.5 also confirmed Flag antibody co-immunostained with Rcor2 antibody and LSD1 in all cells of the neocortex (Supplementary Fig. 4a) and the protein localization was similar to the staining results using Rcor2 antibody (Fig. 1d). Immunoprecipitation (IP) followed by western blot analysis revealed that Rcor2-Flag and LSD1 could interact with each other in Rcor2^Flag^ brains (Supplementary Fig. 4b), which may function in a large complex containing multiple components as previous studies^37^,^38^. All results above suggest the expression of Flag in Rcor2^Flag^ mice could indicate the endogenous expression of Rcor2, which serves as a co-repressor of LSD1 complex in the mouse embryonic brains.

Using the Flag M2 antibody, we micro-dissected Rcor2^Flag^ brains at E13.5 to isolate neocortex followed by chromatin IP sequencing (ChIP-seq) analysis (Supplementary Fig. 4c) and identified 1,994 Rcor2-binding peaks in genome-wide scale. Further analysis showed Rcor2-binding sites are mostly (47.6%) enriched in distal intergenic regions, followed by introns and promoters, the ratios of which are 31.1% and 17.2% respectively (Fig. 4c). Binding motif analysis by Multiple EM for Motif Elicitation demonstrates enrichments of AG-rich motifs of Rcor2-binding regions (Fig. 4d). Gene Ontology (GO) analysis of Rcor2 occupancy regions showed significant (*P*<0.01) enrichment of terms related to transcription, cell fate commitment, cell migration and synaptic organization (Fig. 4e), suggesting Rcor2 may directly regulate the transcriptional activity of specific genes involved in neurogenesis during cortical development. In addition, significant (*P*<0.01) enrichment of terms related to Hedgehog signalling could also be observed in GO analysis of Rcor2 occupancy (Fig. 4e), indicating Rcor2 may primarily target genes related to Shh signalling pathway. We also performed ChIP-seq analysis in the dissected Rcor2^Flag^ neocortex at E13.5 using LSD1 antibody. Besides terms related to regulation of transcription, chromatin organization and neural tube development, GO analysis of LSD1 targets also revealed significant (*P*<0.01) enrichment of Hedgehog signalling (Fig. 4f). Moreover, we could observe enhanced H3K4me1 signal in most regulatory regions of Shh pathway-related genes compared with all genes on Rcor2 knockout by H3K4me1 ChIP-seq analysis (Fig. 4g), further suggesting that Rcor2 and LSD1 form a complex, playing roles in Shh signalling pathway regulation.

Detailed investigation of the binding profiles revealed that Rcor2 and LSD1 co-occupied the core promoter regions of *Dlx2* and the upstream regulatory regions of *Shh* (Fig. 4h), as well as the promoters of *Dlx5* and *Ascl1* (Supplementary Fig. 4d), all of which are important for Shh signalling pathway. Coupled with H3K4me1 ChIP-seq results, we found that the regulatory regions of the above genes occupied by Rcor2 and LSD1 were with significantly reduced or even absent H3K4me1 enrichments in Rcor2^Flag^ neocortex (Fig. 4h and Supplementary Fig. 4d). On depletion of Rcor2, although LSD1 enrichments were dramatically decreased, these regions mentioned above exhibited significantly increased H3K4me1 signals (Fig. 4h and Supplementary Fig. 4d), suggesting that Rcor2 may directly target the upstream regulatory regions and recruit LSD1 complex to executive demethylation on H3K4 sites of these genes. ChIP–qPCR analysis further validated the ChIP-seq results above, which showed significant (*P*<0.001) enrichments of Rcor2 in the core promoter regions or enhancers of genes mentioned above in Rcor2^Flag^ neocortex at E13.5 (Fig. 4i and Supplementary Fig. 4e). We also tested whether Rcor1 could functionally substitute for Rcor2 to repress the target genes. ChIP–qPCR analysis using Rcor1 antibody showed no significant enrichments of Rcor1 on Shh signalling-related genes (Supplementary Fig. 4f), further suggesting that Rcor1 may target distinct genes from Rcor2 to regulate cortical development. Taken together, our ChIP-seq results of Rcor2^Flag^ and Rcor2^cko^ neocortex demonstrate that Rcor2 working as a co-repressor of LSD1 complex could regulate the transcriptional activities of genes involved in neurogenesis and, more importantly, directly target genes important for Shh signalling pathway, such as *Dlx2* and *Shh*.

### Shh pathway is highly activated in Rcor2-depleted cortex

**Figure 5:**
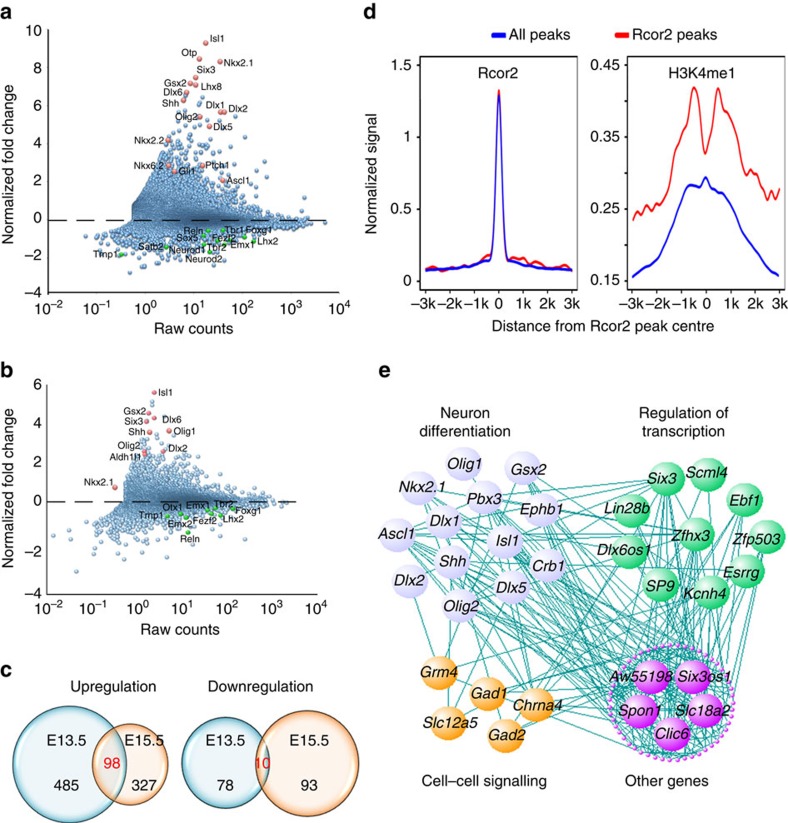
Genome-wide expression changes on Rcor2 depletion in mouse developing cortex by RNA-seq analysis. (**a**,**b**) Scatter plot analysis of genome-wide expression profiles of Rcor2^cko^ versus Rcor2^fl/fl^ samples at E13.5 (**a**) and E15.5 (**b**). Dots above or below the dash line indicate upregulated or downregulated genes on Rcor2 depletion, respectively. Red dots or green dots highlight the significantly differentially expressed genes on Rcor2 depletion. Raw counts (*x* axis); gene expression fold changes on Rcor2 depletion (*y* axis). (**c**) Venn diagrams of upregulated genes (left) and downregulated genes (right) in Rcor2^cko^ samples compared with Rcor2^fl/fl^ samples. (**d**) The profiles of Rcor2 and H3k4me1 enrichments analysed in ChIP-seq results shown in Fig. 3 in regulatory regions of genome-wide scale (red) and of the upregulated genes (purple) according to RNA-seq results. (**e**) Correlation network of overlapped upregulated genes in both E13.5 and E15.5 samples. Lines indicate the correlations between two connected genes with *R*>0.55. Genes were analysed by GO analysis and divided into different categories.

Next, to categorize gene expression changes due to the dysfunction of Rcor2, we performed RNA-sequencing (RNA-seq) to analyse the genome-wide changes on Rcor2 depletion at E13.5 and E15.5, respectively, in developing cortex (Supplementary Fig. 4c). Expression profile of Rcor2 showed RNA peaks of exons between the two *LoxP* sites were totally absent in Rcor2^cko^ cortex at E13.5 and E15.5, indicating successful excision of exons by Cre recombinase (Supplementary Fig. 5a). By comparing gene expression levels in wild-type and knockout samples at the same developmental stage, depletion of Rcor2 predominantly resulted in upregulation of genes, which further confirms its function as a transcriptional co-repressor (Fig. 5a,b). Importantly, we found significant upregulation of *Dlx2*, *Shh*, *Ptch1*, *Gli1*, *Nkx2.1*, *Gsx2* and *Ascl1* at E13.5 (Fig. 5b) and activation of *Dlx2*, *Shh, Nkx2.1* and *Gsx2* at E15.5 (Fig. 5c) when Rcor2 was knocked out, suggesting that Shh signalling pathway is upregulated in both E13.5 and E15.5 samples due to the lack of Rcor2. In addition, genes involved in cortical neurogenesis, such as *Emx1*, *Tbr2*, *Trnp1*, *Foxg1* and *Reln* (Fig. 5a,b), were significantly downregulated on Rcor2 depletion, further suggesting Rcor2's function in cortical neurogenesis regulation.

In the RNA-seq results, 563 genes (485 upregulated and 78 downregulated) at E13.5 and 420 genes (327 upregulated and 93 downregulated) at E15.5 were differentially expressed on Rcor2 depletion as compared with the control samples (*P*<0.01, fold changes >1.5; Fig. 5d). GO term analysis of downregulated genes in E13.5 samples showed a significant (*P*<0.01) enrichment of terms related to neuron differentiation (for example, neuron differentiation, neuron projection development, morphogenesis involved in neuron differentiation, axonogenesis and forebrain development) in the 78 downregulated genes (Supplementary Fig. 5b), similar to the enrichment of terms in E15.5 samples (Supplementary Fig. 5c). Notably, genes involved in chromatin assembly or nucleosome organization were significantly downregulated on Rcor2 depletion at E15.5 stage (Supplementary Fig. 5c), indicating Rcor2 may regulate gene activity involved in neurogenesis by affecting chromatin structure.

Coupled with the aforementioned ChIP-seq results (Fig. 4), we compared both Rcor2-Flag and H3K4me1 enrichment profiles in the regulatory regions of the upregulated genes shown in RNA-seq results with those of genome-wide scale. As the enrichments of Rcor2-Flag in the regulatory regions of upregulated genes could coincide well with those of genome-wide scale, the enrichments of H3K4me1 distributed near the regions of Rcor2 occupancy (however, absent from the Rcor2 peaks in the regulatory regions of genes; Fig. 5d), further suggesting that Rcor2 directly regulate these genes' activities by recruiting LSD1 complex, which would tolerate no methyl modifications on H3K4 sites.

Among the 98 overlapping upregulated genes (Fig. 5c), expression correlation analysis exhibited that 68 gene expressions were positively correlated with other genes with a cutoff *R*-value (correlation coefficient) as 0.55, in which 13 genes were highly related to neuron differentiation, 10 genes were involved in transcription regulation and 5 genes were categorized to cell–cell signalling (Fig. 5e and Supplementary Table S1), indicating that Rcor2 might regulate transcription, neuron differentiation and fate specification. Taken together, both ChIP-seq and RNA-seq results strongly demonstrate that Rcor2 could directly target genes involved in Shh signalling pathway, such as *Shh* and *Dlx2*, to repress their transcriptional activities during cortical neurogenesis.

**Figure 6:**
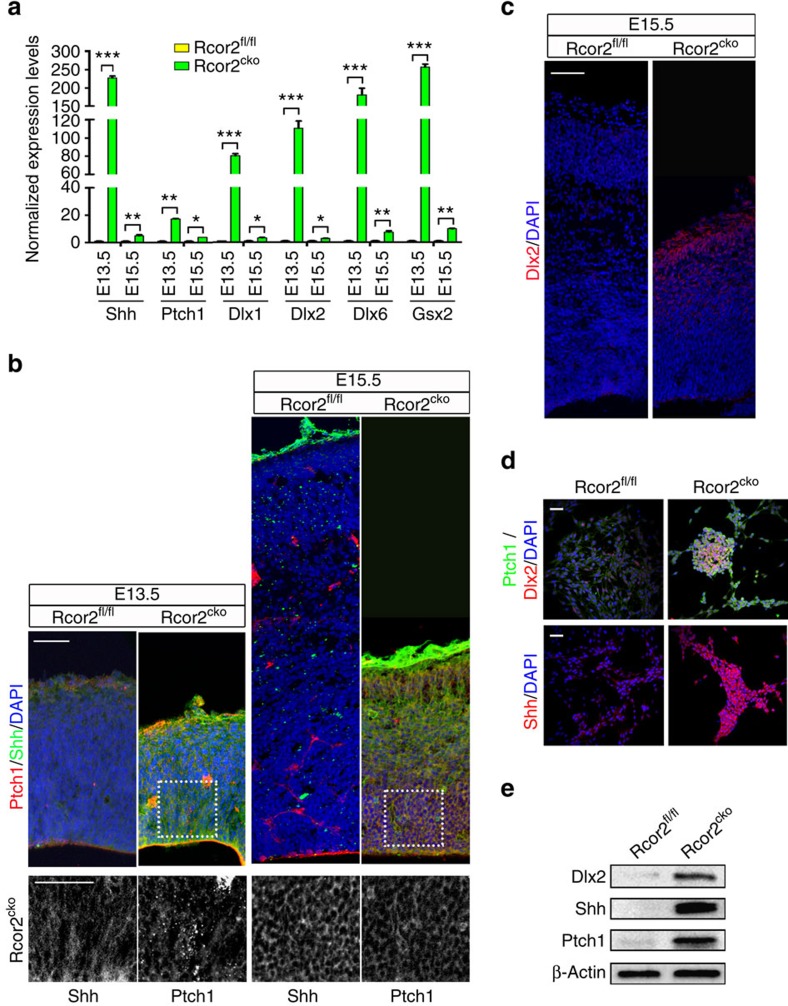
Rcor2 regulates Shh signalling pathway during cortical development. (**a**) qPCR analysis of the expression of genes related to the Shh signalling pathway in the cortex of Rcor2^fl/fl^ and Rcor2^cko^ brains during development. Significant upregulation of these genes on Rcor2 depletion is noteworthy. Transcripts were normalized to Rcor2^fl/fl^ group. Data are shown as mean±s.d., *t*-test, *P<0.05, **P<0.01 and ***P<0.001, *n*=3. (**b**) Confocal images of Shh and Ptch1 expressions in Rcor2^fl/fl^ and Rcor2^cko^ cortex. Enhanced Shh and Ptch1 signals are observed in Rcor2^cko^ compared with Rcor2^fl/fl^ neocortex at E13.5 and E15.5. Insets show high-magnification images of the outlined regions. Scale bars, 50 μm. (**c**) Dlx2 expression in Rcor2^fl/fl^ and Rcor2^cko^ cortex detected by immunofluorescence analysis at E15.5. Dlx2^+^ cells were observed in the neocortex on Rcor2 depletion. Scale bars, 50 μm. (**d**) Confocal images of Shh, Ptch1 and Dlx2 expressions in *in-vitro*-cultured Rcor2^cko^ NPCs. Scale bar, 20 μm. (**e**) Western blot analysis of expression levels of Dlx2, Shh and Ptch1 in Rcor2^fl/fl^ and Rcor2^cko^ cortex at E15.5. β-Actin is used as an endogenous control.

Given the direct enrichments of Rcor2 on molecules involved in Shh signalling (Figs 4 and 5), we validated the expression of genes involved in Shh signalling pathways on Rcor2 depletion. RT–qPCR analysis of both *in-vivo* neocortical samples and *in-vitro*-cultured NPCs revealed significant activation of Shh signalling pathway on Rcor2 depletion (Fig. 6a and Supplementary Fig. 6a). Immunostaining results showed the signals of Shh and its receptor Ptch1, which maintained low levels in Rcor2^fl/fl^ cortex during development as reported^19^, were both significantly enhanced with localization on the membrane of cortical cells on Rcor2 depletion (Fig. 6b). Strikingly, we found enrichment of Dlx2 signals at E15.5 in the neocortex on Rcor2 depletion (Fig. 6c), consistent with ChIP-seq and RNA-seq data. The staining observations above were validated by immunostaining in cultured Rcor2^cko^ NPCs *in vitro*, which also exhibited significantly enhanced expression of Shh, Ptch1 and Dlx2 (Fig. 6d). In addition, western blot analysis also confirmed the upregulation of the above genes related to Shh signalling on Rcor2 knockout during cortical development (Fig. 6e). Taken together, both *in vivo* and *in vitro* data above strongly suggest that Rcor2 plays an important role in repression of Shh signalling pathway.

Previous studies demonstrated that Shh, as a classic morphogen, plays important roles in dorsal–ventral specification during CNS development^19^,^39^. Next, we checked whether abnormally activated Shh signalling caused by Rcor2 depletion affected dorsal–ventral gene expression pattern during cortical development. Similar to our RNA-seq results (Fig. 5b), quantitative expression analysis also showed significant downregulation of dorsal marker genes such as *Emx1* and *Emx2*, and dramatically upregulated ventral marker genes including *Nkx2.1*, *Dlx6*, *Olig2* and so on in E15.5 neocortex on Rcor2 depletion (Supplementary Fig. 6b). Besides the defects in excitatory neuron production on Rcor2 knockout during cortical development (Fig. 3 and Supplementary Fig. 3a), we also observed enriched expressions of interneuron markers, Calretinin and Somatostatin, in Rcor2^cko^ dorsal cortex at E15.5 by immunostaining (Supplementary Fig. 6c). These results strongly suggest that Rcor2 function in repression of Shh signalling pathway, thereby specifying dorsal–ventral gene expression patterns during cortical development, resulting in ectopic generation of cells that might be inhibitory neurons.

Although using Nestin-driven conditional knockout mice is a typical choice for the central nerve system research, however, to further study Rcor2 function in neocortical development we generated Rcor2^fl/fl^Emx1^Cre^ (Rcor2^Emx^) mice to specifically knock out Rcor2 in the neocortex^40^. We observed similar phenotype in Rcor2^Emx^ developing neocortex (Supplementary Fig. 7), excluding the possibility that our observations in Rcor2^cko^ neocortex is caused by the side effects of Nestin-driven Rcor2 depletion and further confirming Rcor2's important roles during neocortical development.

### Inhibition of Shh partially rescues Rcor2-depletion defects

**Figure 7:**
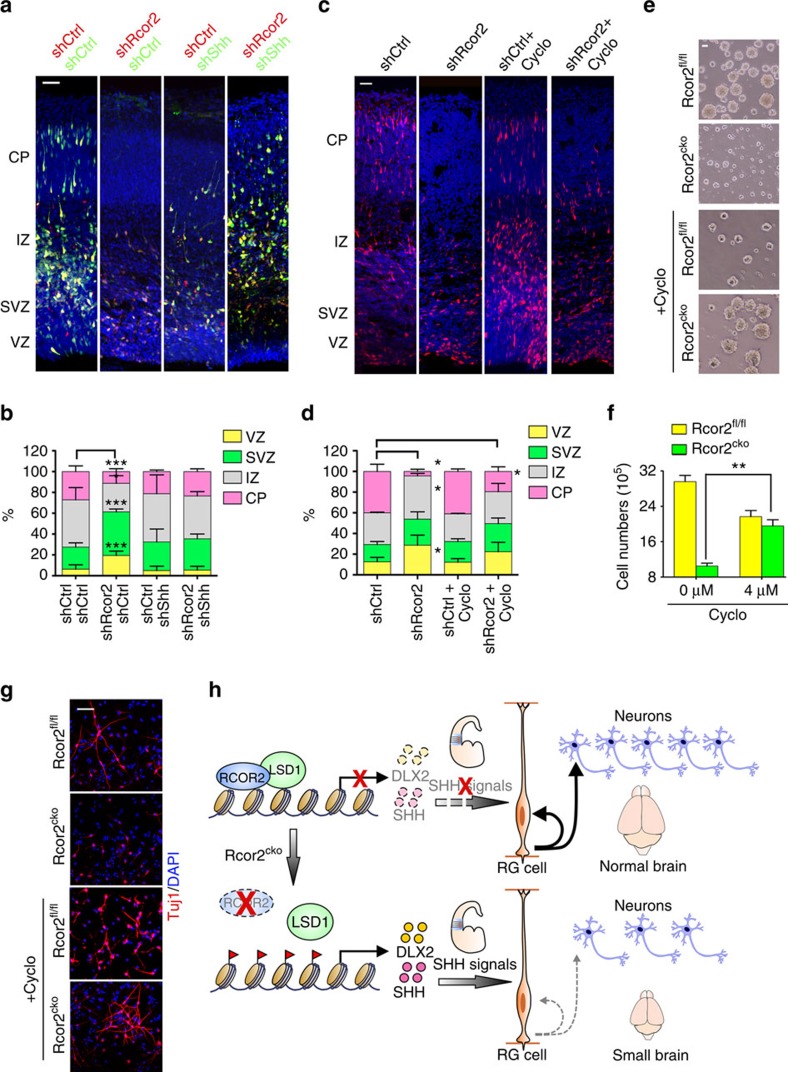
Rcor2 regulates cortical development by inhibition of Shh. (**a**) Knockdown of Rcor2 impairs cortical neurogenesis, which can be partially rescued by knockdown of Shh during cortical development. *In-utero* electroporation with RFP-shControl (red)/GFP-shControl (green), RFP-shRcor2 (red)/GFP-shControl (green), RFP-shControl (red)/GFP-shShh (green) or RFP-shRcor2 (red)/GFP-shShh (green) plasmids was performed at E13.5. Cerebral sections were fixed and imaged at E16.5. VZ, ventricular zone; SVZ, subventricular zone; CP, cortical plate. Scale bar, 50 μm. (**b**) Quantification of the percentage of RFP^+^/GFP^+^ cells in different regions of the developing cortex after electroporation shown in **a**. Data are shown as mean±s.e.m., *t*-test, **P<0.01 and ***P<0.001, *n*=3 individual experiments. (**c**) Inhibition of Shh by Cyclopamine can partially rescue neurogenesis defects caused by Rcor2 downregulation during cortical development. Rcor2 was knocked down at the lateral ventricle in the brain by *in-utero* electroporation with RFP-shRcor2 plasmids at E13.5. Cerebral sections were collected at E14.5 and then treated with cyclopamine to inhibit Shh activity for 48 h. Scale bar, 50 μm. (**d**) Quantification of the percentage of RFP^+^ cells in different regions of the developing neocortex after knockdown of Rcor2 or inhibition of Shh shown in **c**. Data are shown as mean±s.e.m., *t*-test, *P<0.05, *n*=3 individual experiments. (**e**) Representative images depicting neurosphere size is partially rescued in the *in-vitro*-cultured Rcor2^cko^ NPCs after treatment with Cyclopamine. Scale bar, 50 μm. (**f**) Histogram depicting cell numbers of *in-vitro*-cultured Rcor2^fl/fl^ and Rcor2^cko^ NPCs with or without Cyclopamine treatment for 48 h. Cells (5 × 10^5^) are seeded initially. Data are shown as mean±s.e.m., *t*-test, **P<0.01, *n*=3. (**g**) Confocal images of Tuj1 expression in the differentiated cells from *in-vitro*-cultured Rcor2^fl/fl^ and Rcor2^cko^ NPCs with or without Cyclopamine treatment. Tuj1 expressions are partially restored in Cyclopamine-treated Rcor2^cko^ cells. Scale bar, 20 μm. (**h**) Model of Rcor2 function in neurogenesis in the developing neocortex. Rcor2 safeguards cortical neurogenesis by recruiting LSD1 complex to the regulatory regions of Dlx2 and Shh genes, to inhibit the Shh pathway activation during development. The absence of Rcor2 leads to inhibition release of these genes and thus ectopic activation of Shh signalling in the developing neocortex, resulting in cortical neurogenesis defects.

Given the profound fact that Rcor2 regulates cortical neurogenesis by repressing Shh and its downstream factor Dlx2 during development, we next asked whether inhibition of Shh could rescue the phenotypes caused by Rcor2 disruption. To test this, both specific shRNAs and inhibitor Cyclopamine^41^ were adopted to inhibit Shh activity in the cortex electroporated with Rcor2 RFP–shRNAs. Electroporation of Shh shRNAs into the shRcor2 neocortex could partially rescue the neurogenesis defects caused by Rcor2 knockdown (Fig. 7a,b). Similarly, Cyclopamine treatment of the shRcor2 neocortex could also relieve such defects (Fig. 7c,d), suggesting that Rcor2 regulates cortical neurogenesis through regulating Shh signalling pathway.

In addition, when adding Cyclopamine into the medium of the *in-vitro*-cultured NPCs for 48 h, the neurosphere sizes of Rcor2^cko^ NPCs were dramatically increased, similar in size to those of wild-type NPCs (Fig. 7e). Cell counting further confirmed about twofold increases in Rcor2^cko^ NPC numbers after Cyclopamine treatment compared with the untreated NPCs (Fig. 7f), suggesting improved proliferative ability of Rcor2^cko^ NPCs on Shh inhibition. Notably, ectopically activated Shh and Ptch1 signals in Rcor2^cko^ NPCs were downregulated and Dlx2^+^ cells decreased on Cyclopamine treatment (Supplementary Fig. 8a), indicating that Rcor2 regulates NPC proliferation through Shh signalling pathway.

Depletion or knockdown of Rcor2 impairs neuronal differentiation of cortical RGCs during development (Figs 2, 3 and 5, and Supplementary Figs 2, 3, 5 and 7). Hence, to further test whether the affected neuronal production caused by Rcor2 disruption can be rescued by Shh inhibition, Cyclopamine was introduced into the differentiation process of *in-vitro*-cultured NPCs. Immunostaining results showed the ratio of Tuj1^+^ cells in the Rcor2^cko^ group was significantly increased on Cyclopamine treatment, similar to those of the Rcor2^fl/fl^ group (Fig. 7g and Supplementary Fig. 8b), suggesting that the impaired neuronal differentiation of Rcor2-depleted NPCs could also be partially rescued by Shh inhibition *in vitro*.

## Discussion

Histone demethylase LSD1/KDM1A can form complexes with different co-repressors in different cell types for fate regulation^42^,^43^,^44^. How specific subunits of LSD1 complex regulate gene activation and repression in brain development remains unclear. So far, three Rcor family genes (*Rcor1*/*CoREST*, *Rcor2* and *Rcor3*) have been reported in mammalian genomes and their orthologous genes have also been found in *Xenopus*, *Drosophilla* and *Caenorhabditis elegans*^45^. Rcor1, as the first characterized Rcor family member, has been extensively investigated in previous reports. However, the roles of Rcor2 and testis-specific Rcor3 remain largely unknown. Using conditional knockout mouse models coupled with CRISPR/Cas9 genome-editing system, our study first identify that Rcor2 inhibits transcriptional activities by facilitating nucleosomal demethylase activities of LSD1 complex on the promoter regions of *Dlx2* and *Shh*, both related to Shh signalling during cortical development. Furthermore, the ectopic activation of Shh signals caused by Rcor2 disruption results in inappropriate gene expression patterns in the developing dorsal neocortex and thus gives rise to defective NSC/NPC proliferation and differentiation, and finally severely impairs cortical neurogenesis and reduces brain size (Fig. 7h).

As previously reported, Shh signalling pathway is essential to CNS development throughout different developmental stages for axes formation^10^,^46^,^47^. In the early developing telencephalon, Shh is mainly expressed in the MGE and PoA, to restrict ventral patterning and to maintain cortical interneuron progenitor identity, while maintaining a low level in the neural stem/progenitor cells and in mature neurons in the dorsal telencephalon^19^,^48^. In our study, we find that the LSD1 co-repressor Rcor2 is enriched in the core promoter regions of several genes downstream of Shh signalling such as *Dlx2*, *Dlx5* and *Ascl1*, and the regulatory region of *Shh* (Fig. 4h and Supplementary Fig. 4d), thus sustaining the low activity of Shh signals during cortical development. In addition, not only the RNA transcripts but also the protein levels of the genes involved in Shh signalling is increased dramatically in the developing neocortex of Rcor2^cko^ brain *in vivo* and cultured Rcor2^cko^ NPCs *in vitro* (Figs 5 and 6, and Supplementary Fig. 6). This is the first evidence to demonstrate Rcor2 as a direct epigenetic regulator of Shh signalling pathway at the transcriptional level.

Disrupting or depleting Rcor2 expression results in RGC division abnormality and cell death (Fig. 2l,m), resulting in reduction of newborn neuron population, neocortex thickness and brain size (Figs 1f,g and 3a–e, and Supplementary Fig. 1d–g). These neurogenesis defects could be partially rescued by inhibiting Shh signals (Fig. 7), indicating that the ectopic Shh expression in the dorsal cortex could be a reason for defective cortical lamination on Rcor2 depletion, resulting in small brains. Interestingly, some research groups have considered that Shh is a mitogen regulating precursor cell proliferation in dorsal brain^15^,^18^. Studies from Shikata *et al*.^49^ have suggested that strong upregulation of Shh signalling activates transition from dorsalized IPCs (Tbr2^+^) to ventralized IPCs (Dlx2^+^), whereas low expression of Shh is required for cortical IPC proliferation in VZ. Moreover, other findings revealed that Shh activation in dorsal cortex convert dorsal/ventral marker gene expression^50^. Consistent with that, our observations here suggested strong expression of Shh in dorsal brain on Rcor2 depletion, resulting in depletion of dorsalized NPCs/IPCs and reduction of pyramidal neurons. In addition, we found an increase of ventralized IPCs and interneurons in the developing neocortex. Combined with the previous studies discussed above, it is suggested that the temporal and spatial expression of Shh is critical for the elaborative regulation of neurogenesis. Rcor2 not only directly regulates transcription of *Shh* but also some other ventral marker genes, such as *Dlx2*, *Dlx5* and *Ascl1* (Fig. 4 and Supplementary Fig 4). GO analysis of RNA-seq and ChIP-seq did not reveal much direct bindings between Rcor2 and promoter regions of neuronal target genes, indicating that the neurogenesis defects and neuronal cell fate changes caused by Rcor2 depletion could be a systematic alteration of dorsal cell characteristics change and telencephalon environment disruption, in which Shh signalling might play a critical role as an effector. Consistent with this, RNA-seq analysis revealed that genes involved in cell migration, cell fate commitment and synapse organization were also changed on Rcor2 depletion, which could be results from the systematic alterations as well. However, we cannot rule out the direct role of Rcor2 in regulating synapse organization and circuits formation.

Previous reports showed that ectopic Shh signalling in dorsal cortex induces expression of ventral marker genes and reduces expressions of dorsal marker genes^49^,^50^. Consistent with these observations, we found that when Shh signalling was abnormally activated on Rcor2 depletion in dorsal telencephalon at E13.5 and E15.5, the Shh receptor Ptch1 and its downstream ventral marker genes were dramatically upregulated, including *Dlx2*, *Nkx2.1*, *Gsx2*, *Ascl1* and so on (Figs 5, 6 and Supplementary Fig. 6). More importantly, we also found that expressions of dorsal marker genes such as *Emx1/2*, *Tbr2, Trnp1, Foxg1* and so on were reduced (Fig. 5 and Supplementary Fig. 6). In addition, we observed increase of interneurons in dorsal neocortex (Supplementary Fig 6c), which could be generated locally due to ectopic expression of ventral genes.

Taken together, our data support a model where Rcor2 directly represses transcription activities of Shh signalling pathway, to guarantee dorsal neocortex neurogenesis. Moreover, our *in-vitro* data recapitulated our *in-vivo* observations, highlighting the striking similarity of epigenetic regulation mechanisms both *in vivo* and *in vitro*. In summary, our findings open an avenue for the understanding of neurogenesis regulation via epigenetic regulatory complexes such as co-repressors in forebrain development.

## Methods

### Aminals

Adult Rcor2^LacZ^ mice (C57BL/6N-Rcor2^tm1a(EUCOMM)Wtsi^) were obtained from the Welcome Trust Sanger Institute Mouse Genetics Projects (Sanger MGP). Rcor2^fl/fl^ mice were generated by crossing Rcor2^LacZ^ mice with Rosa26^Flp^ mice. Rcor2^cko^ mice were produced by crossing Rcor2^fl/fl^ mice with Nestin^Cre^ mice. Rcor2^fl/fl^Emx1^Cre^ (Rcor2^Emx^) mice were generated by crossing Rcor2^fl/fl^ mice with Emx1^Cre^ mice. All mice had free access to food and water. All experiments were performed in accordance with the University of Health Guide for the Care and Use of Laboratory Animals and were approved by the Biological Research Ethics Committee of Tongji University.

### X-gal staining

Embryos at E11.5 were perfused with 4% paraformaldehyde in PBS (pH 7.4) followed by staining with 1 g l^−1^ X-Gal (Invitrogen).

### Nissl staining and immunostaining of brain sections

Embryos at the desired developmental stages were removed and transcardially perfused with ice-cold PBS (pH 7.4) followed by 4% paraformaldehyde in PBS (pH 7.4). Brains were dissected out and coronal sections were prepared using a vibratome (Leica Microsystems). For Nissl staining, brain slides were stained in 0.1% Cresyl violet for 3 min after dehydration and rehydration. Images were taken by Leica SCN400 (Leica Microsystems). For immunostaining, brain slides were subjected to antigen retrieval, followed by permeabilization (0.1% Triton X-100 in PBS), blocking (10% donkey serum in PBS) and antibody incubation. Images were acquired using a confocal laser scanning microscope (FV1000MPE-BX61WI, Olympus) and were analysed using FluoView (Olympus), Imaris (Bitplane) and Photoshop (Adobe Systems). Primary antibodies used were as follows: Rcor2 (1:50, catalogue number NBP1-74099, Novus Biological); Nes (1:300, catalogue number Rat-401, DSHB); Pax6 (1:100, DSHB); Sox2 (1:500, catalogue number sc-17319, Santa Cruz); BrdU (1:500, catalogue number ab6326, Abcam); Ki67 (1:300, catalogue number ab9260, Millipore); Satb2 (1:500, catalogue number ab34735, Abcam); Olig2 (1:200, catalogue number ab109186, Abcam); Shh (1:100, catalogue number sc-33943, Santa Cruz); Ptch1 (1:100, catalogue number sc-6149, Santa Cruz); Dlx2 (1:500, catalogue number ab117546, Abcam); Nkx2.1 (1:300, catalogue number ab76013, Abcam); Tbr1 (1:500, catalogue number ab31940, Abcam); Dcx (1:500, catalogue number ab18723, Abcam); Tbr2 (1:500, catalogue number ab23345, Abcam); Flag M2 (1:1,000, catalogue number F1804, Sigma); Map2 (1:500, catalogue number ab32454, Abcam); and Tuj1 (1:1,000, catalogue number mms-435p, Convance). Secondary antibodies used were as follows: goat or donkey anti-mouse or anti-rabbit Alexa-546-, Alexa-488- and Alexa-647-conjugated antibodies (1:500, Invitrogen). DNA was stained with 49,6-diamidino-2-phenylindole (1:10,000, catalogue number D1306, Invitrogen).

### Western blotting

For western blotting analysis, cortical cells were lysed in lysis buffer (20 mM Tris pH 7.0, 1% Triton X-100, 0.5% NP-40, 250 mM NaCl, 3 mM EDTA and protease inhibitor cocktail). Proteins were separated by SDS–PAGE and transferred onto a nitrocellulose membrane. After membranes were blocked with 5% milk for 30 min, they were probed with various primary antibodies overnight at 4 °C, followed by incubation with secondary antibodies for 1 h at room temperature and visualized with enhanced chemiluminescence reagent (Thermo Scientific). Images have been cropped for presentation. Full-size images are presented in Supplementary Fig. 9.

### cRNA probe synthesis and *in situ* hybridization

cRNA probe was synthesized by forward primer 5′- GAGAGCCTCTGCCATCCA -3′ and reverse primer 5′- GCCAGAGAGATCCAGCCA -3′. For *in situ* hybridization, all operations were carried out under RNase-free conditions. Frozen sections of 20-μm thickness were hybridized with cRNA probes at a final concentration of 400 ng ml^−1^ overnight at 68 °C in hybridization solution (50% formamide, 10% dextran sulfate, 0.2% tRNA (Invitrogen), 1 × Denhardts solution (from a 50 × stock; Sigma), 1 × salt solution (containing 0.2 M NaCl, 0.01 M Tris, 5 mM NaH_2_PO_4_, 5 mM Na_2_HPO_4_, 5 mM EDTA pH 7.5)). After the sections were washed, alkaline phosphatase-coupled anti-digoxigenin Fab fragments were applied. For visualization of the labelled cRNAs, the sections were incubated in the dark in nitrobluetetrazolium/5-bromo-4-chloro-3-indolyl phosphate solution (Roche). Images were taken by Leica SCN400 (Leica Microsystems).

### Plasmid preparation and *in-utero* electroporation

Rcor2 shRNA sequences were cloned into pSicoR-RFP vector, Rcor2 sequences were cloned into pMX-FLAG-HA-IRES-GFP vector and Shh shRNA sequences were cloned into pLL3.7 vector, as previously described^51^. *In-utero* electroporation was performed as previously described^51^. In brief, a timed pregnant CD-1 mouse or Rcor2^fl/fl^ mouse at E13.5 was anaesthetized, the uterine horns were exposed and 1 μl of plasmid DNA (1–3 μg μl^−1^) mixed with Fast Green (Sigma) was manually microinjected through the uterus into the lateral ventricle, using a beveled and calibrated glass micropipette (Drummond Scientific). For electroporation, five 50-ms pulses of 40–50 mV with a 950-ms interval were delivered across the uterus with two 9-mm electrode paddles positioned on either side of the head (ECM830, BTX). After the procedure, the uterus was placed back in the abdominal cavity and the wound was surgically sutured. The mouse was then placed in a 28 °C recovery incubator under close monitoring until it recovered and resumed normal activity. Related primer sequences were listed in Supplementary Table 1.

### Time-lapse imaging of cortical slice culture

Cortical slice cultures were prepared and time-lapse imaging was acquired as previously described^8^,^51^. About 1 day after *in-utero* electroporation, embryos were removed and the brain was extracted into ice-cold artificial cerebrospinal fluid containing the following: 125 mM NaCl, 5 mM KCl, 1.25 mM NaH_2_PO_4_, 1 mM MgSO_4_, 2 mM CaCl_2_, 25 mM NaHCO_3_, 20 mM glucose pH 7.4 and 310 mOsm l^−1^. Brains were embedded in 3% low-melting agarose in artificial cerebrospinal fluid and sectioned at 400 mm using a vibratome (Leica Microsystems). Brain slices were transferred on to a slice culture insert (Millicell) in a glass-bottom Petri dish (MatTek Corporation) with culture medium containing (by volume): 66% Basal Medium Eagle (BME), 25% Hanks, 5% fetal bovine serum, 1% N2, 1% penicillin/streptomycin/glutamine (Invitrogen) and 0.66% D-(1)-glucose (Invitrogen). Cultures were maintained in a humidified incubator at 37 °C with constant 5% CO_2_ supply. Twenty-four hours later, Petri dishes with slice cultures were transferred to an inverted microscope FV1000MPE-IX81ZDC (Olympus) and imaged every 20 min for about 2–3 days. Images were analysed by FluoView (Olympus), Imaris (Bitplane) and Photoshop (Adobe Systems).

### Reverse transcriptase–quantitative PCR

Total RNAs of Rcor2^fl/fl^ and Rcor2^cko^ brains at desired developmental stages were extracted using TRIzol reagent (Invitrogen) according to the manufacturer's instructions. Complementary DNA synthesis was performed with the M-MLV Reverse Transcriptase Kit (Promega) following the manufacturer's instructions. Quantitative RT–PCR was performed using a SYBR Green Premix Ex Taq II (Takara) and signals were detected with ABI7500 Real-Time PCR System (Applied BioSystems). Related primer sequences were listed in Supplementary Table 1 and Gapdh was used as endogenous control.

### RNA-seq and data analysis

For RNA-seq analysis, a timed pregnant Rcor2^fl/+^Nes^Cre^ mouse crossed with Rcor2^fl/+^Nes^Cre^ male mouse at E13.5 were anaesthetized. Embryos were removed separately and genotyping was performed to identify the Rcor2^fl/fl^ and Rcor2^cko^ pups. Cortical slices of Rcor2^fl/fl^ and Rcor2^cko^ embryos were prepared as described above. The VZ–SVZ regions were dissected separately and RNA was isolated using RNeasy Mini kit (Qiagen) according to the manufacturer's instructions. RNA-seq was performed by Biodynamic Optical Imaging Center at Peking University. For data analysis, sequencing reads were mapped with default settings using Tophat software v2.0.3 to the mouse genome reference (UCSC mm9). Differential gene expression analysis and scatter plotting were performed with Cuffdiff and R as previously described^52^.

### ChIP-seq and ChIP–qPCR

Rcor2^Flag^ mice were first generated by knocking in 3 × Flag sequences adjacent to the start codon ATG of *Rcor2* gene locus by CRISPR/Cas9. Embryonic cortex were isolated from Rcor2^Flag^ and Rcor2^cko^ embryos at E13.5, respectively. Cross-linking, chromatin isolation, sonication and IP were performed using MAGnify Chromatin Immunoprecipitation System (Invitrogen) following the manufacturer's instructions. Flag M2 antibody (1:1,000, catalogue number F1804, Sigma) and LSD1 antibody (1:1000, catalogue number 17721, Abcam) were used in the ChIP assays. Eluted DNA was used either for qPCR analysis as described above by using input DNA as the endogenous control or high-throughput sequencing. The pair-end sequencing was performed using Illumina Genome Analyzer IIx and Sequencing Kits (Version 5) after liabrary preparation. Illumina CASAVA pipeline v1.8.0 was used for the following sequence extraction and filtering.

## Additional information

**Accession codes:** All sequencing-derived data reported in this study have been deposited in NCBI's Gene Expression Omnibus (GEO) under accession number GSE75040.

**How to cite this article**: Wang, Y. *et al*. LSD1 co-repressor Rcor2 orchestrates neurogenesis in the developing mouse brain. *Nat. Commun.* 7:10481 doi: 10.1038/ncomms10481 (2016).

## Supplementary Material

Supplementary Information Supplementary Figures 1-9 and Supplementary Table 1

## References

[b1] Caviness V. S. Jr. & Rakic P. Mechanisms of cortical development: a view from mutations in mice. Annu. Rev. Neurosci. 1, 297–326 (1978) . 386903 10.1146/annurev.ne.01.030178.001501

[b2] Anderson S. A., Marin O., Horn C., Jennings K. & Rubenstein J. L. Distinct cortical migrations from the medial and lateral ganglionic eminences. Development 128, 353–363 (2001) . 11152634 10.1242/dev.128.3.353

[b3] Anderson S. A., Kaznowski C. E., Horn C., Rubenstein J. L. & McConnell S. K. Distinct origins of neocortical projection neurons and interneurons in vivo. Cereb. Cortex 12, 702–709 (2002) . 12050082 10.1093/cercor/12.7.702

[b4] Molyneaux B. J., Arlotta P., Menezes J. R. & Macklis J. D. Neuronal subtype specification in the cerebral cortex. Nat. Rev. Neurosci. 8, 427–437 (2007) . 17514196 10.1038/nrn2151

[b5] Rakic P. The radial edifice of cortical architecture: from neuronal silhouettes to genetic engineering. Brain Res. Rev. 55, 204–219 (2007) . 17467805 10.1016/j.brainresrev.2007.02.010 PMC2203611

[b6] Wonders C. P. & Anderson S. A. The origin and specification of cortical interneurons. Nat. Rev. Neurosci. 7, 687–696 (2006) . 16883309 10.1038/nrn1954

[b7] Xu Q. . Sonic hedgehog signaling confers ventral telencephalic progenitors with distinct cortical interneuron fates. Neuron 65, 328–340 (2010) . 20159447 10.1016/j.neuron.2010.01.004 PMC2868511

[b8] Wang X., Tsai J. W., LaMonica B. & Kriegstein A. R. A new subtype of progenitor cell in the mouse embryonic neocortex. Nat. Neurosci. 14, 555–561 (2011) . 21478886 10.1038/nn.2807 PMC3083489

[b9] Puelles L. & Rubenstein J. L. Forebrain gene expression domains and the evolving prosomeric model. Trends Neurosci. 26, 469–476 (2003) . 12948657 10.1016/S0166-2236(03)00234-0

[b10] Fuccillo M., Joyner A. L. & Fishell G. Morphogen to mitogen: the multiple roles of hedgehog signalling in vertebrate neural development. Nat. Rev. Neurosci. 7, 772–783 (2006) . 16988653 10.1038/nrn1990

[b11] Liu A. & Joyner A. L. Early anterior/posterior patterning of the midbrain and cerebellum. Annu. Rev. Neurosci. 24, 869–896 (2001) . 11520921 10.1146/annurev.neuro.24.1.869

[b12] Campbell K. Dorsal-ventral patterning in the mammalian telencephalon. Curr. Opin. Neurobiol. 13, 50–56 (2003) . 12593982 10.1016/s0959-4388(03)00009-6

[b13] Guillemot F. Cellular and molecular control of neurogenesis in the mammalian telencephalon. Curr. Opin. Cell Biol. 17, 639–647 (2005) . 16226447 10.1016/j.ceb.2005.09.006

[b14] Sur M. & Rubenstein J. L. Patterning and plasticity of the cerebral cortex. Science 310, 805–810 (2005) . 16272112 10.1126/science.1112070

[b15] Dahmane N. . The Sonic hedgehog-Gli pathway regulates dorsal brain growth and tumorigenesis. Development 128, 5201–5212 (2001) . 11748155 10.1242/dev.128.24.5201

[b16] Gulacsi A. & Anderson S. A. Shh maintains Nkx2.1 in the MGE by a Gli3-independent mechanism. Cereb. Cortex 16, (Suppl 1): i89–i95 (2006) . 16766713 10.1093/cercor/bhk018

[b17] Komada M. Sonic hedgehog signaling coordinates the proliferation and differentiation of neural stem/progenitor cells by regulating cell cycle kinetics during development of the neocortex. Congenit. Anom. (Kyoto) 52, 72–77 (2012) . 22639991 10.1111/j.1741-4520.2012.00368.x

[b18] Komada M. . Hedgehog signaling is involved in development of the neocortex. Development 135, 2717–2727 (2008) . 18614579 10.1242/dev.015891

[b19] Sousa V. H. & Fishell G. Sonic hedgehog functions through dynamic changes in temporal competence in the developing forebrain. Curr. Opin. Genet. Dev. 20, 391–399 (2010) . 20466536 10.1016/j.gde.2010.04.008 PMC2991106

[b20] Fong A. P. . Genetic and epigenetic determinants of neurogenesis and myogenesis. Dev. Cell 22, 721–735 (2012) . 22445365 10.1016/j.devcel.2012.01.015 PMC3331915

[b21] MuhChyi C., Juliandi B., Matsuda T. & Nakashima K. Epigenetic regulation of neural stem cell fate during corticogenesis. Int. J. Dev. Neurosci. 31, 424–433 (2013) . 23466416 10.1016/j.ijdevneu.2013.02.006

[b22] Ma D. K. . Epigenetic choreographers of neurogenesis in the adult mammalian brain. Nat. Neurosci. 13, 1338–1344 (2010) . 20975758 10.1038/nn.2672 PMC3324277

[b23] Hirabayashi Y. & Gotoh Y. Epigenetic control of neural precursor cell fate during development. Nat. Rev. Neurosci. 11, 377–388 (2010) . 20485363 10.1038/nrn2810

[b24] Okano H. & Temple S. Cell types to order: temporal specification of CNS stem cells. Curr. Opin. Neurobiol. 19, 112–119 (2009) . 19427192 10.1016/j.conb.2009.04.003

[b25] Hsieh J. & Gage F. H. Chromatin remodeling in neural development and plasticity. Curr. Opin. Cell Biol. 17, 664–671 (2005) . 16226449 10.1016/j.ceb.2005.09.002

[b26] Ballas N. & Mandel G. The many faces of REST oversee epigenetic programming of neuronal genes. Curr. Opin. Neurobiol. 15, 500–506 (2005) . 16150588 10.1016/j.conb.2005.08.015

[b27] Shi Y. . Histone demethylation mediated by the nuclear amine oxidase homolog LSD1. Cell 119, 941–953 (2004) . 15620353 10.1016/j.cell.2004.12.012

[b28] Qureshi I. A., Gokhan S. & Mehler M. F. REST and CoREST are transcriptional and epigenetic regulators of seminal neural fate decisions. Cell Cycle 9, 4477–4486 (2010) . 21088488 10.4161/cc.9.22.13973 PMC3048046

[b29] Yao H. . Corepressor Rcor1 is essential for murine erythropoiesis. Blood 123, 3175–3184 (2014) . 24652990 10.1182/blood-2013-11-538678 PMC4023423

[b30] Andres M. E. . CoREST: a functional corepressor required for regulation of neural-specific gene expression. Proc. Natl Acad. Sci. USA 96, 9873–9878 (1999) . 10449787 10.1073/pnas.96.17.9873 PMC22303

[b31] Lunyak V. V. . Corepressor-dependent silencing of chromosomal regions encoding neuronal genes. Science 298, 1747–1752 (2002) . 12399542 10.1126/science.1076469

[b32] Abrajano J. J. . Corepressor for element-1-silencing transcription factor preferentially mediates gene networks underlying neural stem cell fate decisions. Proc. Natl Acad. Sci. USA 107, 16685–16690 (2010) . 20823235 10.1073/pnas.0906917107 PMC2944745

[b33] Fuentes P., Canovas J., Berndt F. A., Noctor S. C. & Kukuljan M. CoREST/LSD1 control the development of pyramidal cortical neurons. Cereb. Cortex 22, 1431–1441 (2012) . 21878487 10.1093/cercor/bhr218

[b34] Yang P. . RCOR2 is a subunit of the LSD1 complex that regulates ESC property and substitutes for SOX2 in reprogramming somatic cells to pluripotency. Stem Cells 29, 791–801 (2011) . 21433225 10.1002/stem.634

[b35] Skarnes W. C. . A conditional knockout resource for the genome-wide study of mouse gene function. Nature 474, 337–342 (2011) . 21677750 10.1038/nature10163 PMC3572410

[b36] Nair V. D. . Involvement of histone demethylase LSD1 in short-time-scale gene expression changes during cell cycle progression in embryonic stem cells. Mol. Cell. Biol. 32, 4861–4876 (2012) . 23028048 10.1128/MCB.00816-12 PMC3497594

[b37] Suva M. L. . Reconstructing and reprogramming the tumor-propagating potential of glioblastoma stem-like cells. Cell 157, 580–594 (2014) . 24726434 10.1016/j.cell.2014.02.030 PMC4004670

[b38] Ding J., Xu H., Faiola F., Ma'ayan A. & Wang J. Oct4 links multiple epigenetic pathways to the pluripotency network. Cell Res. 22, 155–167 (2012) . 22083510 10.1038/cr.2011.179 PMC3252465

[b39] Marti E. & Bovolenta P. Sonic hedgehog in CNS development: one signal, multiple outputs. Trends Neurosci. 25, 89–96 (2002) . 11814561 10.1016/s0166-2236(02)02062-3

[b40] Gorski J. A. . Cortical excitatory neurons and glia, but not GABAergic neurons, are produced in the Emx1-expressing lineage. J. Neurosci. 22, 6309–6314 (2002) . 12151506 10.1523/JNEUROSCI.22-15-06309.2002 PMC6758181

[b41] Taipale J. . Effects of oncogenic mutations in Smoothened and Patched can be reversed by cyclopamine. Nature 406, 1005–1009 (2000) . 10984056 10.1038/35023008

[b42] Mosammaparast N. . The histone demethylase LSD1/KDM1A promotes the DNA damage response. J. Cell Biol. 203, 457–470 (2013) . 24217620 10.1083/jcb.201302092 PMC3824007

[b43] Toffolo E. . Phosphorylation of neuronal Lysine-Specific Demethylase 1LSD1/KDM1A impairs transcriptional repression by regulating interaction with CoREST and histone deacetylases HDAC1/2. J. Neurochem. 128, 603–616 (2014) . 24111946 10.1111/jnc.12457

[b44] Liu L. . Histone lysine-specific demethylase 1 (LSD1) protein is involved in Sal-like protein 4 (SALL4)-mediated transcriptional repression in hematopoietic stem cells. J. Biol. Chem. 288, 34719–34728 (2013) . 24163373 10.1074/jbc.M113.506568 PMC3843083

[b45] Lakowski B., Roelens I. & Jacob S. CoREST-like complexes regulate chromatin modification and neuronal gene expression. J. Mol. Neurosci. 29, 227–239 (2006) . 17085781 10.1385/JMN:29:3:227

[b46] Traiffort E., Angot E. & Ruat M. Sonic Hedgehog signaling in the mammalian brain. J. Neurochem. 113, 576–590 (2010) . 20218977 10.1111/j.1471-4159.2010.06642.x

[b47] Machold R. . Sonic hedgehog is required for progenitor cell maintenance in telencephalic stem cell niches. Neuron 39, 937–950 (2003) . 12971894 10.1016/s0896-6273(03)00561-0

[b48] Xu Q., Wonders C. P. & Anderson S. A. Sonic hedgehog maintains the identity of cortical interneuron progenitors in the ventral telencephalon. Development 132, 4987–4998 (2005) . 16221724 10.1242/dev.02090

[b49] Shikata Y. . Ptch1-mediated dosage-dependent action of Shh signaling regulates neural progenitor development at late gestational stages. Dev. Biol. 349, 147–159 (2011) . 20969845 10.1016/j.ydbio.2010.10.014

[b50] Huang X., Litingtung Y. & Chiang C. Ectopic sonic hedgehog signaling impairs telencephalic dorsal midline development: implication for human holoprosencephaly. Hum. Mol. Genet. 16, 1454–1468 (2007) . 17468181 10.1093/hmg/ddm096

[b51] Wang X. . Asymmetric centrosome inheritance maintains neural progenitors in the neocortex. Nature 461, 947–955 (2009) . 19829375 10.1038/nature08435 PMC2764320

[b52] Gao Y. . Replacement of Oct4 by Tet1 during iPSC induction reveals an important role of DNA methylation and hydroxymethylation in reprogramming. Cell Stem Cell 12, 453–469 (2013) . 23499384 10.1016/j.stem.2013.02.005

