# Supplementary material for: LSD1 co-repressor Rcor2 orchestrates neurogenesis in the developing mouse brain
Source: Nat Commun. 2016 Jan 22;7:10481. doi: 10.1038/ncomms10481 (PMC4736047; doi:10.1038/ncomms10481)
Supplement: Supplementary Information — Supplementary Figures 1-9 and Supplementary Table 1 [file ncomms10481-s1.pdf]

Supplementary Figure 1

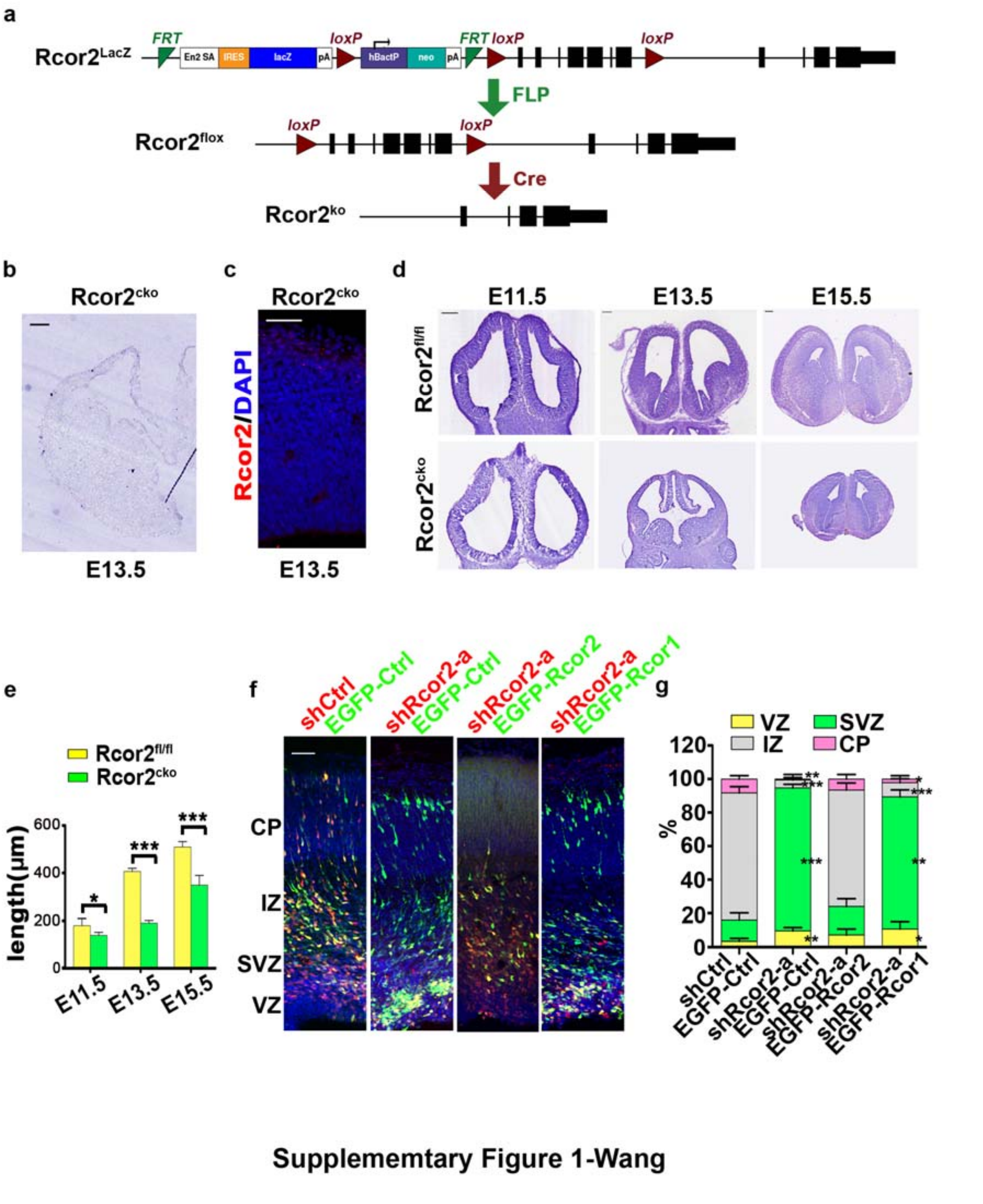

Supplementary Fig. 1: Rcor2 expresses in the central nervous system (CNS), regulating cortical development.

(a) Schematic of Rcor2-trapped mice construction strategy. A LacZ reporter cassette, flanked by two FRT sequences, is inserted ahead of the first exon to disrupt Rcor2 expression in the Rcor2^{LacZ} mice. Two LoxP sites are inserted between the first several exons in these mice. The LacZ reporter cassette can be removed by Flippase splicing, and the Rcor2 gene can be

knocked out in different tissues by tissue-specific Cre recombinase splicing.

(b) Representative image of *in situ* hybridization in $Rcor2^{cko}$ brain sections at E13.5. Scale bar, 100μm.

(c) Representative immunostaining of $Rcor2^{cko}$ brain section at E13.5 using Rcor2 antibody. Scale bar, 50μm.

(d) Nissl staining of $Rcor2^{fl/fl}$ and $Rcor2^{cko}$ brains at different stages. Representative images of $Rcor2^{fl/fl}$ and $Rcor2^{cko}$ brain slices show cortical hypoplasia with reduced thickness in $Rcor2^{cko}$ neocortex. Scale bar, 200μm.

(e) Histograms depicting quantitative changes in cortical thickness between $Rcor2^{fl/fl}$ and $Rcor2^{cko}$ developing neocortex. Y-axis, lengths of cortical thickness. Data are shown as mean ± S.E.M., t test, * $p<0.05$, *** $p<0.001$, n=3 separate staining from 3 independent brains.

(f) Representative images of E16.5 cerebral sections electroporated with RFP-shControl (red)/ EGFP-Control (green), RFP-shRcor2 (red) /EGFP-Control (green), RFP-shRcor2 (red) /EGFP-mRcor2 (green), or RFP-shRcor2 (red) /EGFP-mRcor1 (green) plasmids at E13.5. VZ, ventricular zone; SVZ, sub-ventricular zone; IZ, intermediate zone; CP, cortical plate. Scale bar, 20μm.

(g) Quantification of the percentage of RFP^+/ $EGFP^+$ cells in different regions of the developing neocortex after electroporation shown as (f). Y-axis, percentage of RFP^+/ $EGFP^+$ cells in different zones. Data are shown as mean ± S.E.M., t test, *$p < 0.05$, ** $p < 0.01$, *** $p < 0.001$, n=3.

Supplementary Figure 2

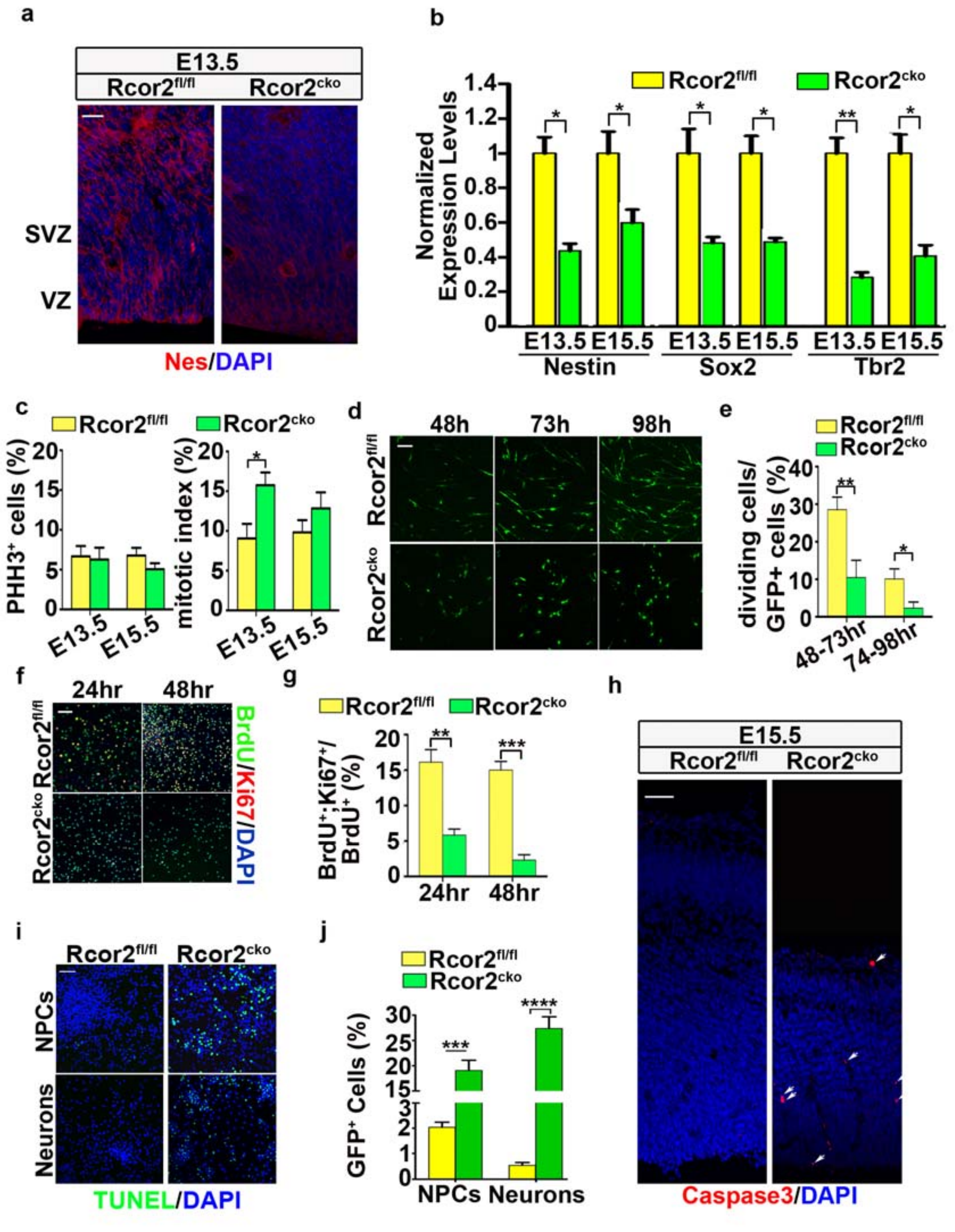

Supplememtary Figure 2 - Wang

Supplementary Fig. 2: Cortical NSC/NPC population and proliferation are affected upon Rcor2 depletion both *in vivo* and *in vitro*.

(a) Confocal images of Nestin immunostaining at E13.5. Sox2 and Tbr2 are dramatically reduced upon Rcor2 depletion. VZ, ventricular zone; SVZ, sub-ventricular zone. Scale bar, 20μm.

(b) RT-qPCR analysis of Nestin, Sox2 and Tbr2 expression levels in both Rcor2$^{fl/fl}$ and Rcor2^{cko}

neocortex at E13.5 and E15.5. Transcripts were normalized to the $Rcor2^{fl/fl}$ group. Data are shown as mean ± S.D., t test, *$p < 0.05$, ** $p < 0.01$, n=3.

(c) Quantification of $PHH3^{+}$ cells (left panel) and mitotic index (right panel) in the VZ/SVZ regions of the developing neocortex as shown in Fig. 2f. Data are shown as mean ± S.E.M., t test, n=3 separate stainings from three independent brains.

(d) Time lapse analysis of cultured $Rcor2^{fl/fl}$ and $Rcor2^{cko}$ NPCs after labeling dividing cells with retro-GFP. Scale bar, 100μm.

(e) Quantification of cell division abilities as shown in (d) is calculated by the percentage of dividing cell number in all GFP^{+} cell number at 48-73 hrs and 74-98 hrs after virus infection in $Rcor2^{fl/fl}$ and $Rcor2^{cko}$ NPCs. Y-axis, ratio of dividing cells among GFP^{+} cells. Data are shown as mean ± S.E.M., t test, *$p < 0.05$, ** $p < 0.01$, n=5 from 3 individual experiments.

(f) Confocal images of BrdU (green) and Ki67 (red) staining in *in-vitro* cultured $Rcor2^{fl/fl}$ and $Rcor2^{cko}$ NPCs 24 hrs and 48 hrs after BrdU incubation. Scale bar, 100μm.

(g) Quantification of the cell cycle exit by percentage of $BrdU^{+}$ and $Ki67^{+}$ NPCs divided by $BrdU^{+}$ cells shown in (f). Data are shown as mean ± S.E.M., t test, **$p < 0.01$, *** $p < 0.001$, n=3.

(h) Confocal images of Caspase 3 staining in $Rcor2^{fl/fl}$ and $Rcor2^{cko}$ cortex at E15.5. Arrows: Caspase 3^{+} cells. Scale bar, 100μm.

(i-j) Representative images of TUNEL staining in $Rcor2^{fl/fl}$ and $Rcor2^{cko}$ NPCs and neurons (i), and quantification of GFP^{+} cells in the TUNEL assays (j). Data are shown as mean± S.E.M., t test, *** $p<0.001$, **** $p<0.0001$, n=3. Scale bar, 50μm.

Supplementary Figure 3:

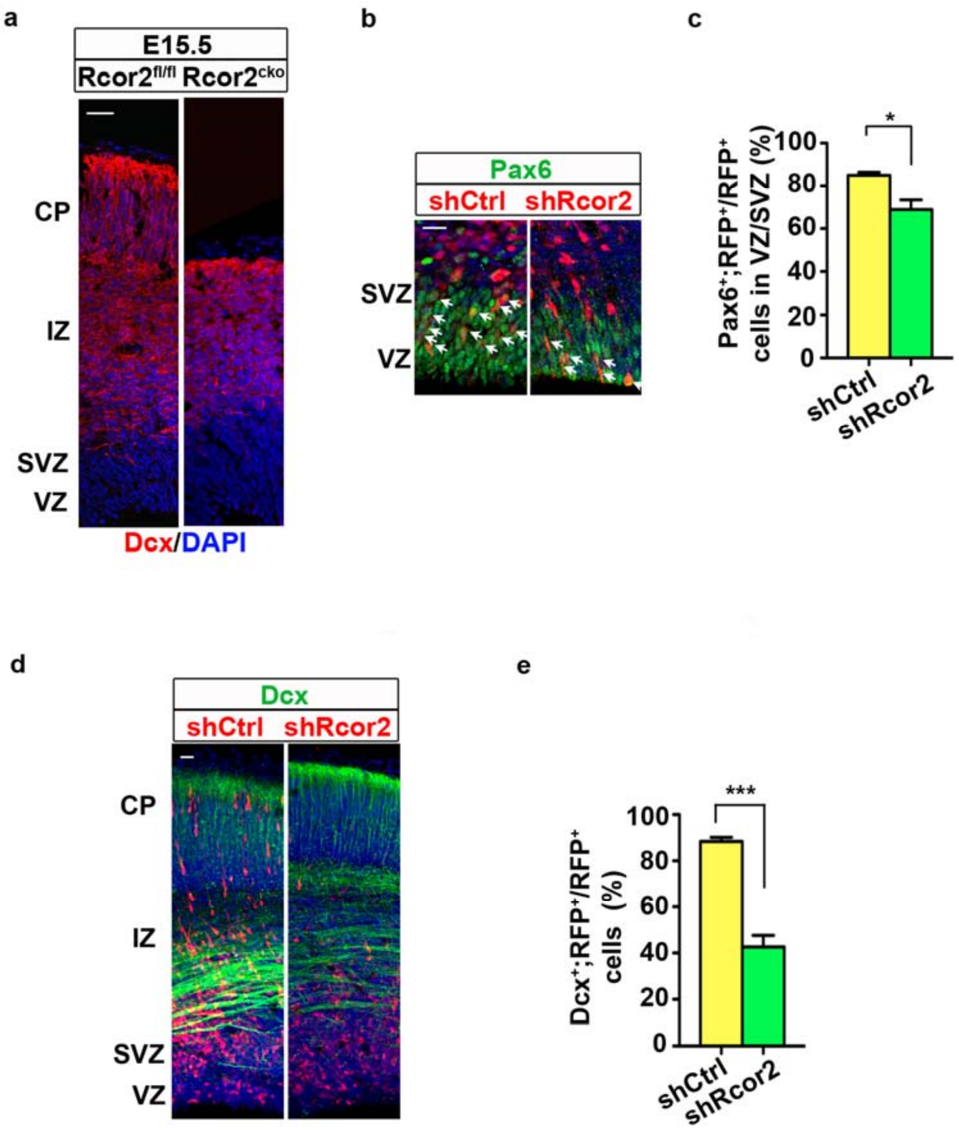

Supplementary Figure 3 - Wang

Supplementary Fig. 3: Neurogenesis is affected upon Rcor2 depletion during cortical development.

(a) Confocal images of Dcx expressions in Rcor2$^{fl/fl}$ and Rcor2^{cko} cortex at E15.5, which exhibit significant reduction upon Rcor2 knock-out. Scale bar, 50 μm.

(b) Representative images of E16.5 cerebral sections electroporated with RFP-shControl (red) or RFP-shRcor2 (red) plasmids at E13.5 and immunostained with Pax6 (green). VZ, ventricular

zone; SVZ, sub-ventricular zone. Arrows: $RFP^{+}Pax6^{+}$ cells; Scale bar, 20μm.

(c) Quantification of the percentage of $RFP^{+}Pax6^{+}/RFP^{+}$ cells in VZ and SVZ as shown in (b). Data are shown as mean ± S.E.M., t test, $*p < 0.05$, n=3.

(d) Representative images of E16.5 cerebral sections electroporated with RFP-shControl (red) or RFP-shRcor2 (red) plasmids at E13.5 and immunostained with Dcx (green). VZ, ventricular zone; SVZ, sub-ventricular zone; IZ, intermediate zone; CP, cortical plate. Scale bar, 20μm.

(e) Quantification of the percentage of $RFP^{+}Dcx^{+}$ /RFP^{+} cells in developing neocortex as shown in (d). Data are shown as mean ± S.E.M., t test, $***p < 0.001$, n=3.

Supplementary Figure 4:

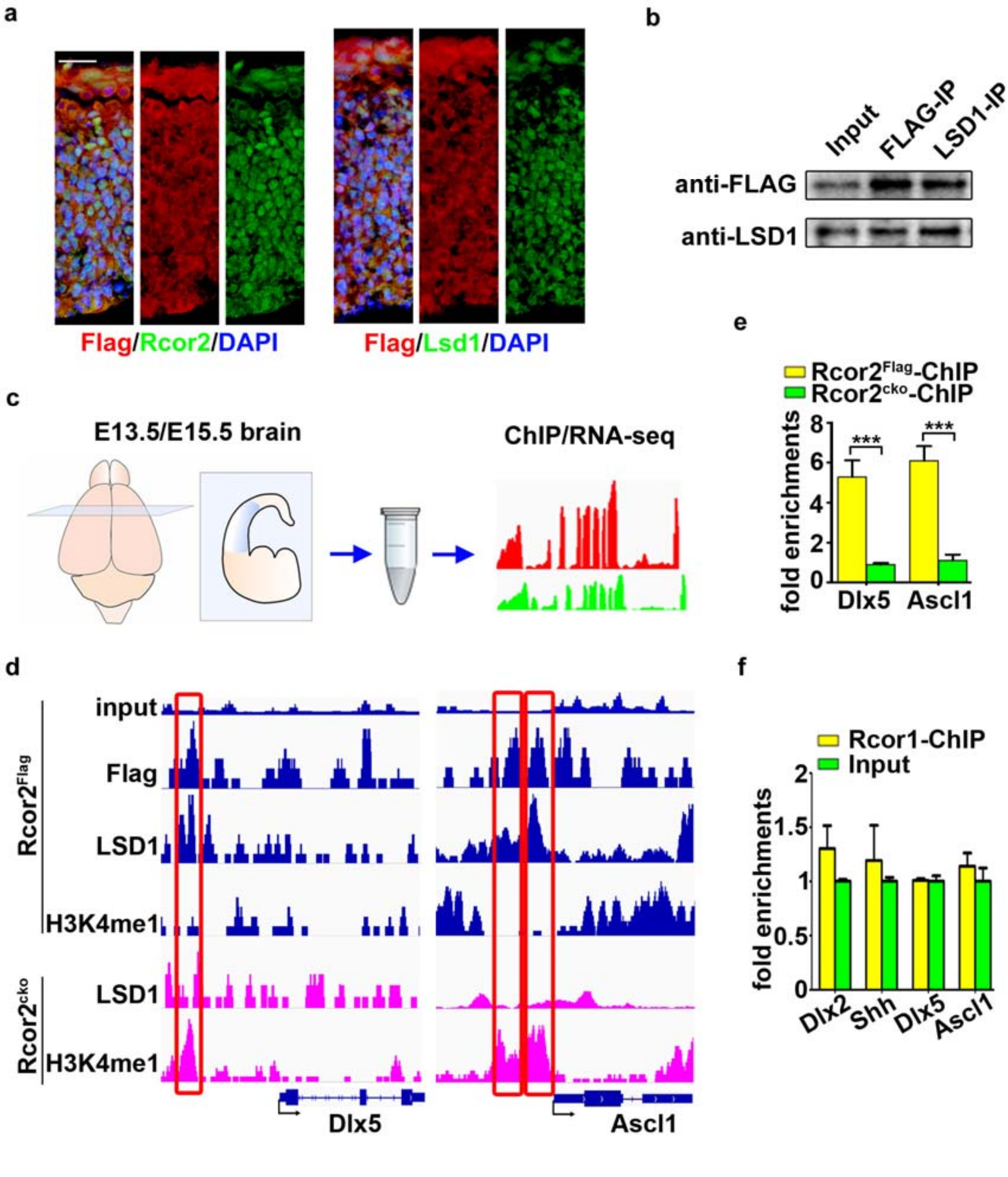

Supplementary Figure 4 - Wang

Supplementary Fig. 4: ChIP-seq analysis of Rcor2 enrichments in genome-wide scale using Rcor2^{Flag} neocortex.

(a) Representative images of Rcor2^{Flag} neocortical section at E11.5 using Flag-M2, Lsd1 and Rcor2 antibodies. Note Flag co-immunostained with RCOR2, were co-expressed with LSD1 in nuclei of neocortex. Scale bar, 50μm.

(b) Western blot analysis for $Rcor2^{Flag}$ brain lysis at E13.5 (input) or the samples derived from anti-FLAG and anti-LSD1 immunoprecipitation using Flag M2 and Lsd1 antibodies.

(c) Schematic of sample preparation for ChIP-seq and RNA-seq using $Rcor2^{fl/fl}$ and $Rcor2^{cko}$ neocortex at E13.5 or E15.5. Embryonic brains were embedded in low-melting-point agarose and sectioned. The tissue marked with light blue color in the brain slice model was isolated for sequencing.

(d) Gene tracks of Rcor2, LSD1 and H3K4me1 enrichments by ChIP-seq analysis at upstream regulatory regions of Dlx5 and Ascl1, which are both related to Shh signaling pathway.

(e) ChIP-qPCR analysis of $Rcor2^{flag}$ and $Rcor2^{cko}$ cortex at E13.5 using specific FLAG-M2 antibody. Note significant enrichments of the Rcor2 at the occupancy region upstream of Dlx5 and Ascl1 gene locus detected in (d) in the $Rcor2^{Flag}$ samples. Y-axis, fold enrichments of Rcor2 occupancy compared to input. Data are shown as mean ± S.D., t test, *** $p < 0.001$, n=3.

(f) ChIP-qPCR analysis of $Rcor2^{fl/fl}$ cortex at E13.5 using specific Rcor1 antibody. No significant enrichments of Rcor1 at Rcor2 binding regions upstream of Dlx2, Shh, Dlx5 and Ascl1 gene locus. Y-axis, fold enrichments of Rcor1 occupancy compared to input. Data are shown as mean ± S.D., t test, n=3.

Supplementary Figure 5:

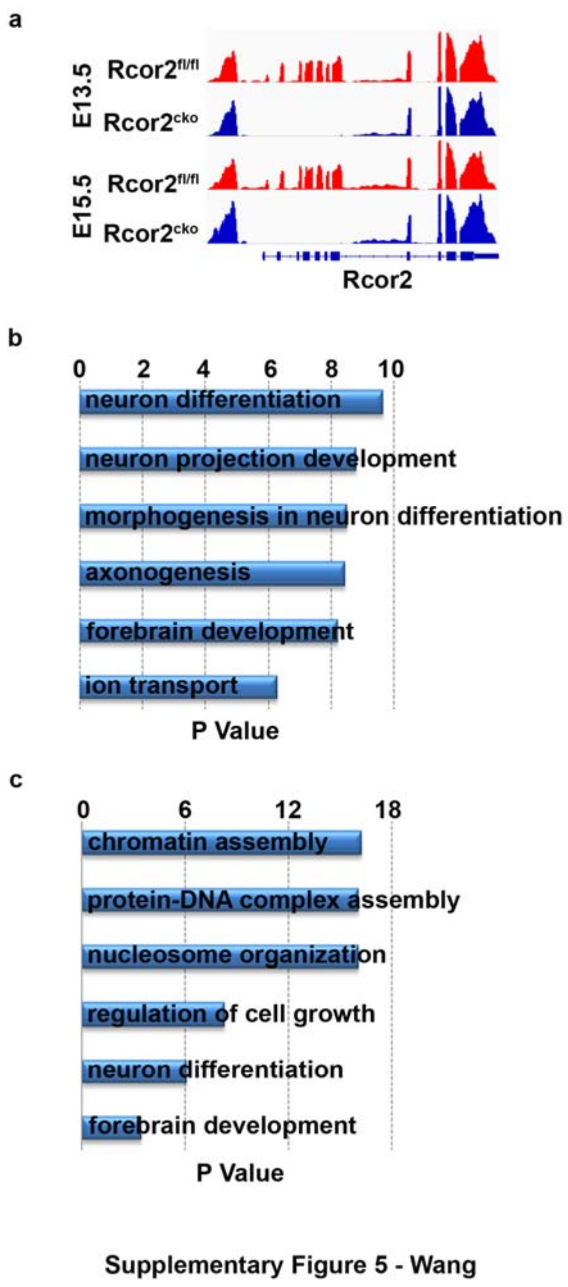

Supplementary Fig. 5: RNA-seq analysis for genome-wide expression changes upon Rcor2 disruption in the developing cortex.

(a) Transcription profiles at Rcor2 gene locus in Rcor2$^{fl/fl}$ and Rcor2^{cko} cortex samples prepared as shown in Supplementary Fig. 4c. Note that RNA peaks of the exons between the two LoxP sites are absent in Rcor2^{cko} E13.5 and E15.5 cortex.

(b) GO analysis for 78 genes down-regulated at E13.5 upon Rcor2 depletion.

(c) GO analysis for 93 genes down-regulated at E15.5 upon Rcor2 depletion.

Supplementary Figure 6:

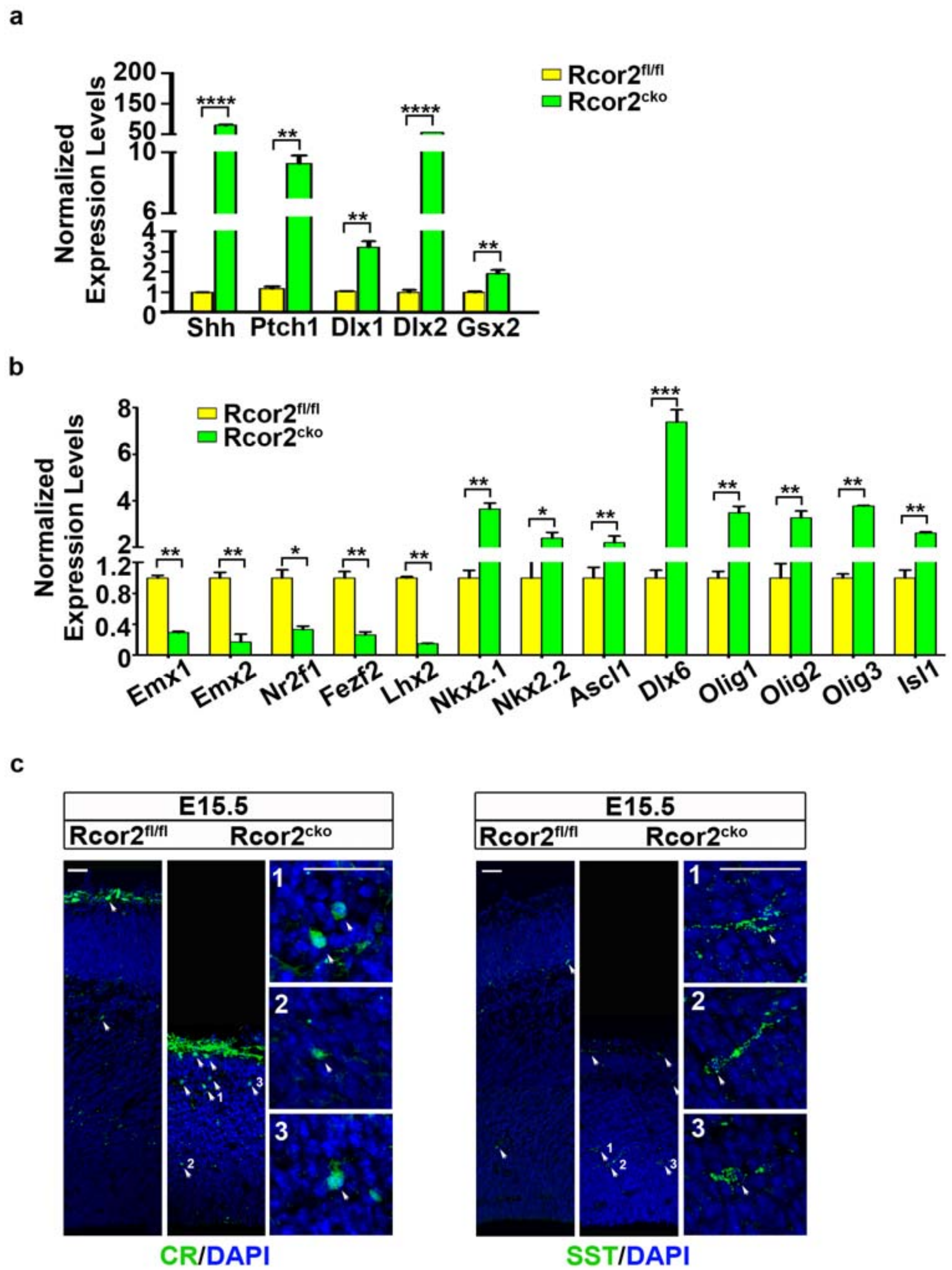

Supplementary Figure 6 - Wang

Supplementary Fig. 6: Rcor2 regulates Shh signaling pathway and dorsal-ventral specification during cortical development.

(a) RT-qPCR analysis of expression of genes involved in Shh signaling pathway in cultured Rcor2$^{fl/fl}$ and Rcor2^{cko} NPCs *in vitro*. Transcripts were normalized to the Rcor2$^{fl/fl}$ group. Data are shown as mean ± S.D., t test, ** p < 0.01, **** p < 0.0001, n=3.

(b) RT-qPCR analysis of expression of genes involved in dorsal-ventral specification in Rcor2$^{fl/fl}$

and Rcor2^{cko} neocortex at E15.5. Transcripts were normalized to the Rcor2$^{fl/fl}$ group. Data are shown as mean ± S.D., t test, *$p < 0.05$, ** $p < 0.01$, *** $p < 0.001$, n=3.

(c) Confocal images of CR (calretinin) and SST (somatostatin) expressions in Rcor2$^{fl/fl}$ and Rcor2^{cko} cortex at E15.5. Enhanced Calretinin and SST signals are observed in Rcor2^{cko} compared to Rcor2$^{fl/fl}$ neocortex. Scale bar, 50 µm. Arrows: CR^{+} or SST^{+} cells.

Supplementary Figure 7:

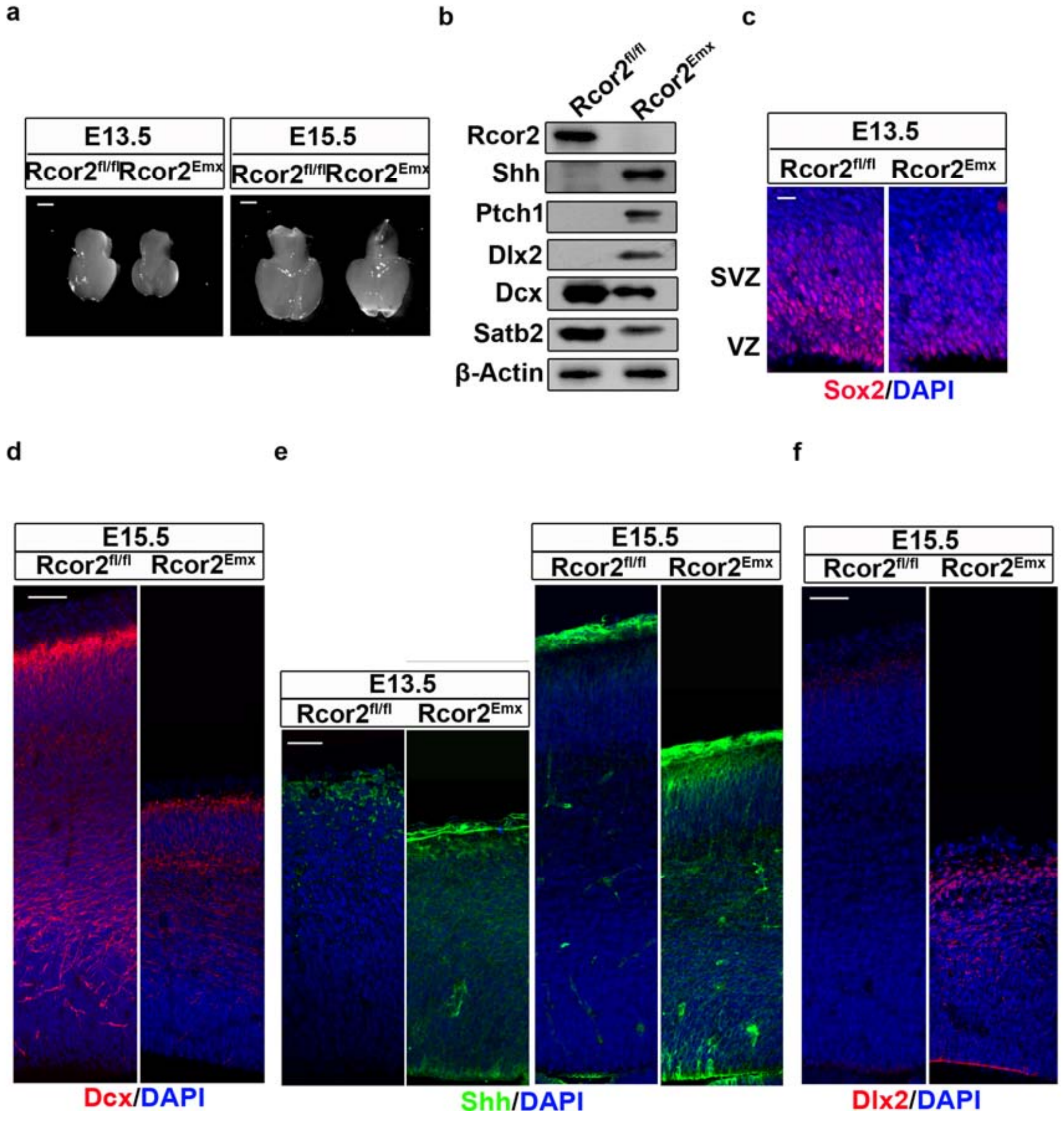

Supplementary Figure 7 - Wang

Supplementary Fig. 7: Characteristics of developing neocortex upon Rcor2 depletion in dorsal neuronal progenitor cells

(a) Representative images of Rcor2$^{fl/fl}$ and Rcor2^{Emx} (generated by Emx1-Cre allele) brain size at different stages of development. Growth retardation is observed in brains upon cortical knockout of Rcor2 at E13.5 and E15.5. Scale bar, 1 mm.

(b) Western blot analysis of expression levels of Rcor2, Shh, Ptch1, Dlx2, Dcx and Satb2 in

Rcor2$^{fl/fl}$ and Rcor2^{Emx} cortex at E15.5. β-Actin is used as an endogenous control.

(c) Immunostaining images of Sox2 in Rcor2$^{fl/fl}$ and Rcor2^{Emx} cortex at E13.5. VZ, ventricular zone; SVZ, sub-ventricular zone. Scale bar, 20μm.

(d-f) Confocal images of Dcx (d), Shh (e) and Dlx2 (f) expressions in Rcor2$^{fl/fl}$ and Rcor2^{Emx} cortex during development, which exhibit significant reduction upon Rcor2 knock-out. Scale bar, 50 μm.

Supplementary Figure 8:

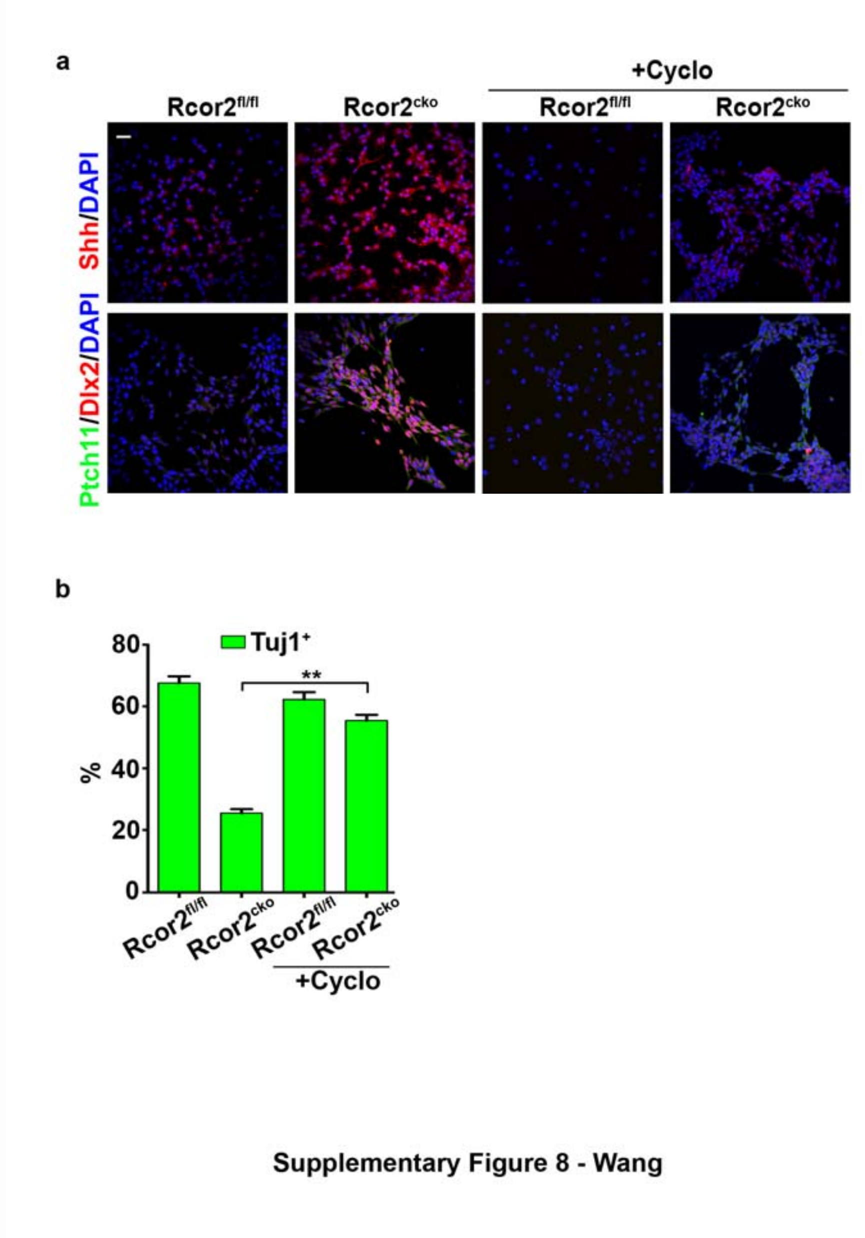

Supplementary Fig. 8: Inhibition of Shh partially rescues the proliferation and neuronal differentiation defects caused by Rcor2 disruption.

(a) Confocal images of Shh, Ptch1 and Dlx2 expressions in *in-vitro* cultured Rcor2$^{fl/fl}$ and Rcor2^{cko} NPCs before and after Cyclopamine treatment for 48 hrs. Inhibition of Shh significantly down-regulates the expression levels of Shh, Ptch1 and Dlx2 in cultured Rcor2^{cko} NPCs. Scale bar, 20 μm.

(b) Quantification of Tuj1^{+} cell ratios in *in-vitro* cultured Rcor2$^{fl/fl}$ and Rcor2^{cko} NPCs before and after Cyclopamine treatment during differentiation as shown in Fig. 7g. Y-axis, proportion of Tuj1^{+} cell. Data are shown as mean ± S.D., t test, ** $p < 0.01$, n=3 independent stainings.

Supplementary Figure 9:

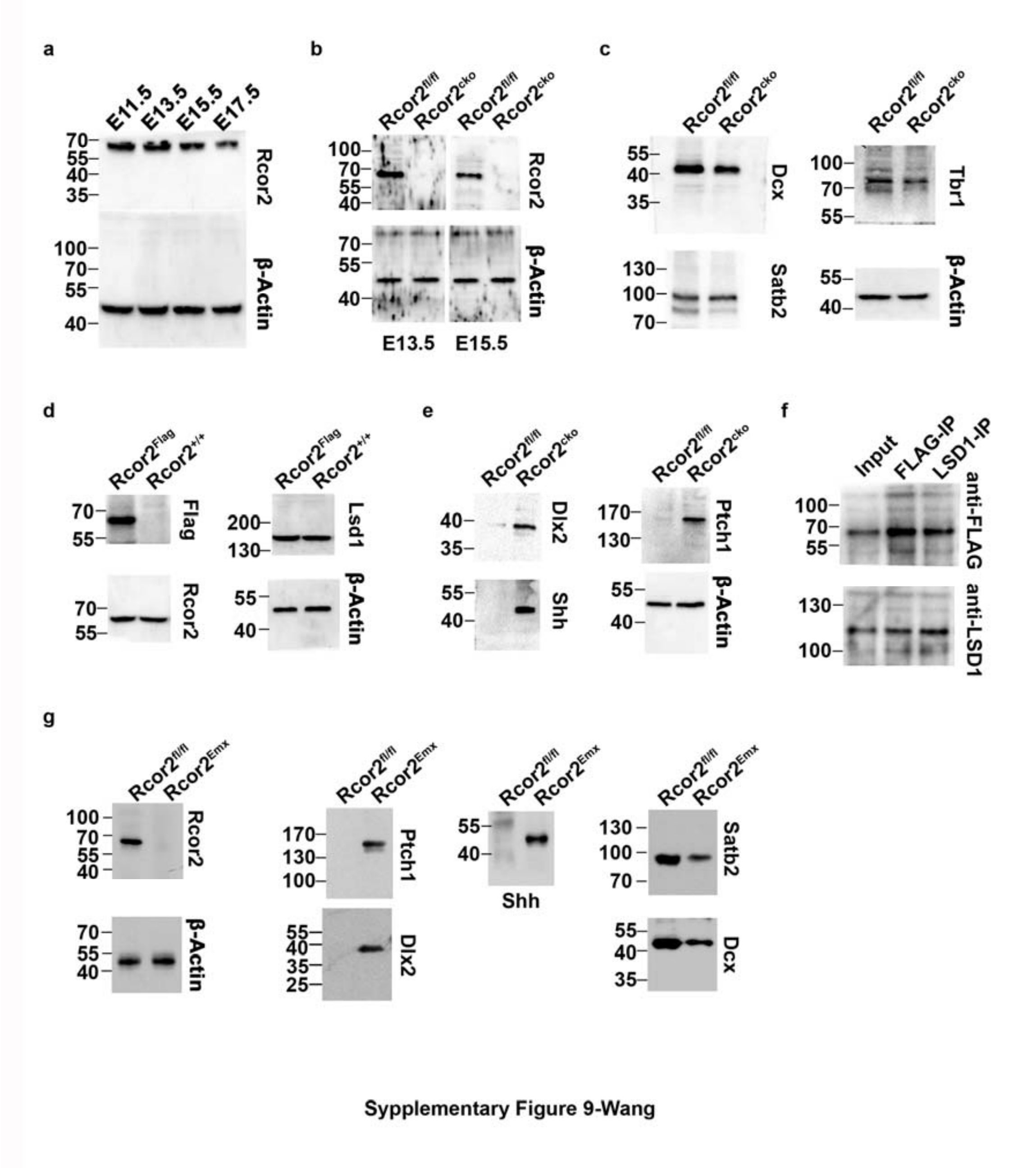

Supplementary Fig. 9: Full-size scans of western blot results shown in main and supplementary figures. Panel a is accordance with Fig. 1b; panel b is accordance with Fig. 1e; panel c is according to Fig. 3c; panel d is according to Fig. 4b; panel e is accordance with Fig. 6e; panel is accordance with Supplementary Fig. 4b.

Supplementary Table 1: Primer sequences

Name	Sequences (5'-3')
shRcor2-a-F	TGCCTGGTGAAGTATTACTATTCAAGAGATAGTAATACTTCAC CAGGCTTTTTTC
shRcor2-a-R	TCGAGAAAAAAGCCTGGTGAAGTATTACTATCTCTTGAATAGT AATACTTCACCAGGCA
shRcor2-b-F	TGTCGAACTAGTGTGATGGATTCAAGAGATCCATCACACTAGT TCGACTTTTTTC
shRcor2-b-R	TCGAGAAAAAAGTCGAACTAGTGTGATGGATCTCTTGAATCC ATCACACTAGTTCGACA
Rcor2-RT-F	GCTGAAGGGAATGCTGGTGTG
Rcor2-RT-R	CAGGGAAGGGAGTGAAGTTGG
Satb2-RT-F	TCTTTTGGGTCTAACCGTCCTAC
Satb2-RT-R	TTTCCTTCACTGACTCCCGTGCT
Map2-RT-F	AGAAACGTTCTTCCCTCCCAA
Map2-RT-R	AAATCCTAACCTGACCCCCCT
Tubb3-RT-F	AGAACAGCAGCTACTTCGTGG
Tubb3-RT-R	TCATCTTCATACATCTCCCCCT
Mapt-RT-F	GGAAATGACGAGAAGAAAGC
Mapt-RT-R	TGTTGGTAGGGATGGGGTGC
Pou4f1-RT-F	AGGTTCGAGTCGCTCACGCTCT
Pou4f1-RT-R	TTTCATCCGCTTCTGCTTCTGT
Syn3-RT-F	GTGAATGGCGACATTGAGATC
Syn3-RT-R	ATTGGCTTGTGGTTGGGGAAA
Syt13-RT-F	CGCCATAGTGTGATTGGGGAA
Syt13-RT-R	GGATTGGTTGGAGTGGAGGTT
Nefl-RT-F	CCAGCCTACTATACCAGCCACGT
Nefl-RT-R	CCTCCTCTTCTGCTTCTCCTTCA
Nefm-RT-F	GCACTACTTGGAACAACAGAA
Nefm-RT-R	ACCTTAACCATCGACGACTCT
Stmn2-RT-F	CCGCGCAACATCAACATCTAC
Stmn2-RT-R	TGCTCCCTCTTCTCTGCCAAC
Omg-RT-F	ATCCTCTTCCACCGCATCCCTT
Omg-RT-R	CTGCCGAGTAGGGTAGGATAA
Nestin-RT-F	CTACCTACCCCTCCCCCATTCAT
Nestin-RT-R	CTCACCTACCTTACGTCCTCGA
Pax6-RT-F	CGTGTGGCTCCCTCTTATTCTTT
Pax6-RT-R	GGCTTGTTATGCGTTTGTGGTTT
Sox2-RT-F	TCTCACTGCCATCTAACACTG
Sox2-RT-R	CTTTTCTTGCTCCACACCTAC
Emx1-RT-F	GTGCTTCGGAATCGCTTCTTT

Emx1-RT-R	TCCAGCTTCTGCCGTTTGTAT
Emx2-RT-F	GTCCCATAAATCCGTTCCTCAA
Emx2-RT-R	GGTTCTTCTCAAAAGCGTGCTC
Nr2f1-RT-F	AAGAAGGAGTTCGTGTGCCG
Nr2f1-RT-R	CTCATTGGAGTGAGTGCGGTTC
Fezf2-RT-F	ACAGAGCAAGCCCACAAGCGA
Fezf2-RT-R	ATCAAGCCAGCGGCACACGAAC
Olig1-RT-F	CCGACGCCAAAGAGGAACAG
Olig1-RT-R	TGCCGAGTAGGGTAGGATAA
Olig2-RT-F	TGGTGTCTAGTCGCCCATCGT
Olig2-RT-R	TCATCTGCTTCTTGTCTTTC
Olig3-RT-F	AGATGAAGAGGTTGGTTGGA
Olig3-RT-R	TGAGCAAGTCCTTGGATTCA
Shh-RT-F	GTTTTCTGGTGATCCTTGCTT
Shh-RT-R	CACTGCTCGACCCTCATAGTG
Ptch1-RT-F	TCTTGCTCTTGGTGTTGGTGTGG
Ptch1-RT-R	CGGGTGTTACTGTGAGGCTCTGT
Isl1-RT-F	CACCCTACCTCTGTCTATTCGC
Isl1-RT-R	GCACCCTTGTTCTGGTTTTACCT
Nkx2.1-RT-F	CGAGCGGCATGAATATGAGTG
Nkx2.1-RT-R	GATCTTGACCTGCGTGGGTGT
Ascl1-RT-F	GAAGATGAGCAAGGTGGAGAC
Ascl1-RT-R	TTGGAGTAGTTGGGGGAGATG
Dlx1-RT-F	GGCTGTGTTTATGGAGTTTGGG
Dlx1-RT-R	CCTGGGTTTACGGATCTTTTTC
Dlx2-RT-F	TGAACGGGAAGCCAAAGAAAGTC
Dlx2-RT-R	TCTGCGAAGGATGCAGAAGTG
Gsx2-RT-F	GCACAAGAAGGAGGGGAAAGG
Gsx2-RT-R	TTAGCCGAGGCAGGGGACAA
Nkx2.2-RT-F	ACGGCAGATGTGTGAGGTTGAC
Nkx2.2-RT-R	TGGGAGGGGTGCTAGTGGGTAA
Dlx6-RT-F	CATCCCCACCTGCCGAAAAAT
Dlx6-RT-R	CACCGATCCAACCTGCGTACA
Lhx2-RT-F	CGAGAAGAAGCTGATGTGTGG
Lhx2-RT-R	AGAGAGCAAGGAATGGGGAGA
Gapdh-RT-F	ACTCCACTCACGGCAAATTC
Gapdh-RT-R	TGGTTCACACCCATCACAAA
Shh-ChIP-F	TAAAGTGCAAAGAGGATGAGGGTC
Shh-ChIP-R	TCGGGAATGTCTGAATCAGGTC
Dlx2-ChIP-F	TACCTTGCAGGGTTGTTTTGA
Dlx2-ChIP-R	TGCGGTGATGTAGGGGTTTAG
Dlx5-ChIP-F	ATACTCTATCCATACCATCC
Dlx5-ChIP-R	TTGTCGTCACAGTCTCCTTC

Ascl1-ChIP-F	CCTGTGCTCTATAAGAAAGG
Ascl1-ChIP-R	CACTGGAACCAAAAGAAACT
Olig2-ChIP-F	TATTACAGACCGAGCCAACAC
Olig2-ChIP-R	CATCCTAAATCCTAGCCACTT
